# Facile Strategy Enabling Fluorine-Free Polyimides with Ultralow Dielectric Loss and Thermal Expansion Close to Copper

**DOI:** 10.1021/acsami.6c02417

**Published:** 2026-04-17

**Authors:** Yu-Hsin Liu, Dula Daksa Ejeta, Yi-Hsin Chang, Wan-Ling Hsiao, Kamani Sudhir K. Reddy, Ching-Hsuan Lin

**Affiliations:** Department of Chemical Engineering, 34916 National Chung Hsing University, Taichung 40227, Taiwan

**Keywords:** polyimide (PI), fluorine-free, dielectric loss, high-frequency, ester dianhydride (TAHQ), m-tolidine, 3,4′-Oxydianiline (3,4-ODA)

## Abstract

Polymer dielectrics for emerging high-speed interconnects must unite a low dielectric constant (*D*_k_) with an ultralow dissipation factor (*D*_f_) while retaining excellent thermal and mechanical robustness. We report a fluorine-free, linear-backbone strategy that pairs an ester-type dianhydride, 1,4-phenylene bis­(1,3-dioxo-1,3-dihydroisobenzofuran-5-carboxylate) (TAHQ), with biphenyl diamines to access ester–ether copolyimides via solution polycondensation and thermal imidization. The homopolyimides from m-tolidine/TAHQ (PI-0) and 3,4-ODA/TAHQ (PI-1) establish that 3,4-ODA is intrinsically favorable for reducing high-frequency loss; building on this, we incorporate 3,4-ODA into the m-tolidine/TAHQ system to obtain copolyimides PI-X (X = mole fraction of 3,4-ODA in the diamine feed). At 10 GHz, the PI-X films achieve *D*_f_ down to 0.0013 with *D*_k_ around 3.1–3.4, giving *D*_f_ × √*D*_k_ values of 0.0024–0.0030 that are lower than those of both parent homopolyimides. The PI-X series also exhibits reduced in-plane coefficients of thermal expansion (CTE). The minimum CTE reaches 11.8 ± 2.8 ppm/°C (*n* = 4), while PI-0.625 shows a CTE of 17.2 ± 1.6 ppm/°C (*n* = 4), which closely matches that of copper (∼17 ppm/°C). This close CTE match is beneficial for mitigating interfacial stress, warpage, and reliability issues in copper-clad laminates and related devices. Without resorting to complex monomer design or multistep diamine syntheses, simple copolymerization with 3,4-ODA simultaneously suppresses high-frequency dielectric loss and lowers CTE, while preserving outstanding thermomechanical performance (*T*_d5%_ = 485–500 °C; tensile strength = 68–157 MPa). These results position TAHQ-based, fluorine-free ester–ether copolyimides incorporating 3,4-ODA and m-tolidine as practical ultralow-loss dielectrics with copper-matched CTEs for high-frequency electronic hardware.

## Graphical abstract

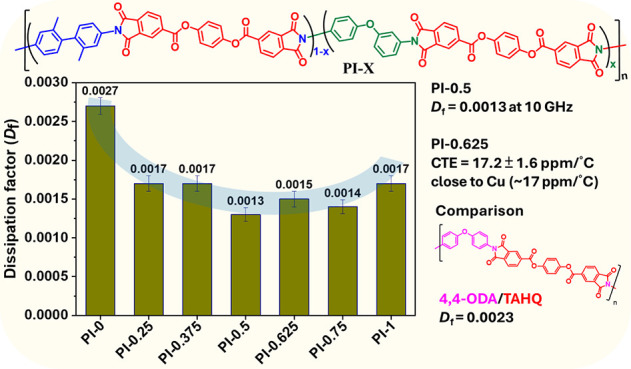

## 1 Introduction

Polyimides (PIs) are a class of high-performance polymers that have long been recognized for their outstanding thermal stability, mechanical strength, and chemical resistance.^1–5^ These attributes make them ideal candidates for use in flexible printed circuit boards (FPCBs), which demand materials capable of maintaining performance under mechanical deformation and elevated temperatures.^6^ With the rapid advancement of 5G and high-frequency communication technologies, the demand for polymer dielectrics that can support high-speed signal transmission with minimal energy loss has become increasingly critical.

The signal propagation loss (*L*) in an integrated circuit is proportional to the frequency, dissipation factor (*D*_f_), and the square root of the dielectric constant (*√D*_k_).^7^ In eq 1, *K* is a constant, *C* is the speed of light, and *f* is the frequency. The propagation loss increases with the frequency. A material with a low *D*_k_ and *D*_f_ value, especially *D*_f_ which is proportional to loss, can compensate for the propagating loss due to the increased frequency in the high-frequency communication. In practice, targets of *D*_f_ ≤ 0.002, together with high thermal stability and low in-plane CTE, define a meaningful performance window for next-generation packaging:

1 $$L=K\times \left(f/C\right)\times D_{f}\times \sqrt{D_{k}}$$

While numerous efforts have been made to reduce *D*_k_ through molecular design and free volume engineering,^8–14^ strategies aimed at lowering *D*_f_ are relatively rare.^15–19^ The dissipation originates from dielectric relaxation processes under alternating electric fields and is closely related to the rotational mobility of polar units within the polymer matrix.^4^ This includes contributions from dipole moment polarization and electronic polarization, which result in energy dissipation.^20^ Consequently, effective *D*_f_ suppression requires design approaches that restrict the rotation of the polarization units and enhance chain rigidity and order, thereby minimizing dielectric loss.

In recent years, liquid crystal polymers (LCPs) have emerged as promising candidates for low-dielectric-loss applications.^21–24^ However, compared with LCPs, PI films offer several advantages, including higher thermal stability, better processability, and lower manufacturing costs. Based on these merits, Hasegawa et al. have reported that the incorporation of ester-containing structures into PI backbones can significantly increase molecular rigidity and lower *D*_f_.^25,26^ Lu et al.^27^ synthesized multiester-containing PIs and systematically investigated their dielectric behavior. They found that increasing the number of ester groups led to a sharp decrease in *D*_f_. Structural analysis using wide-angle X-ray diffraction (WAXD), polarized optical microscopy (POM), and molecular dynamics simulations indicated that the ester groups promoted an ordered morphology and efficient chain organization. POM provided qualitative information about crystalline morphology and optical texture, whereas WAXD and molecular dynamics simulations provided insight into chain packing and intermolecular organization, which contributed to the low dielectric loss. Nonetheless, it often required multistep synthesis to prepare ester-containing diamines such as A_2_EB and A_3_EB in that work.^27^ Furthermore, the ester-containing diamines tend to exhibit low reactivity due to the electron-withdrawing nature of the ester functionalities. In addition, the full-ester PIs have been reported as brittle due to excessive rigidity.^27^ Chern et al. reported that incorporating symmetrical *tert*-butyl groups into a dietheramine improved dielectric *D*_f_ relative to asymmetrical *tert*-butyl substitution.^28^ However, the corresponding symmetrical *tert*-butyl diesteramine homopolyimide exhibited poor solubility and could not be realized. Copolymerizing TAHQ with the *tert*-butyl diesteramine and m-tolidine overcame solubility limitations and yielded a copolyimide with a lower *D*_f_ (0.0036) than the homopolyimide based on m-tolidine/BPDA (0.0049), indicating that judicious comonomer selection can preserve low-loss behavior while improving processability. Chen et al. reported LCPI-4, a liquid-crystal-like PI from 2,2′-bis­(trifluoromethyl)­benzidine TFMB/TAHQ, delivering a *D*_f_ value of 0.0018 (literature) at 10 GHz.^29^ They also reported LCPI-3 from m-tolidine/TAHQ, but the *D*_f_ value increased to 0.00395. This highlights the effectiveness of fluorinated structures in minimizing the dielectric loss. Hsu et al. developed a series of TFDB/NPDA, a naphthalene-based poly­(ester imide)­s, by replacing the central phenylene unit with a rigid naphthalene moiety and found that this structural modification led to a significant reduction in dielectric loss. Specifically, *D*_f_ decreased from 0.0032 for TFDB/TAHQ to 0.0017 for TFDB/NPDA. This improvement was attributed to the increased molecular rigidity and extended conjugation of the naphthalene ring, which effectively suppressed dipolar relaxation processes at high frequencies.^2^ However, the cost for TFDB synthesis is high and the fluorinated building blocks raise the concerns of per- and polyfluoroalkyl substances (PFAS), which are highly persistent, can bioaccumulate, and face tightening regulations across regions.^30–34^

These pressures motivate fluorine-free PI designs that retain low polarization and moisture uptake while approaching the ultralow-loss benchmark set by LCPI-4. To align with evolving PFAS regulations while retaining high-frequency performance, fluorine-free strategies are urgently needed for next-generation, low-loss PI dielectrics. Lu et al.^35^ further demonstrated that incorporating simple ester and ether linkages into the PI backbone yielded PIs with crystalline domains and ultralow *D*_f_. The 4,4-ODA/TAHQ polyimide shows a low *D*_f_ value of 0.0018 (at 10 GHz). This confirmed that molecular linearity and crystallinity are key design principles for reducing dielectric loss in PI systems. Figure 1 presents the single-crystal structure of TAHQ; crystallographic data and refinement details are provided in Tables S1–S2. The molecule adopts an elongated, rod-like conformation, underscoring the linearity and rigidity of the dianhydride. Such a topology is advantageous for low-loss dielectrics, as increased backbone rigidity can suppress high-frequency segmental relaxations that contribute to *D*_f_.

**Figure 1:**
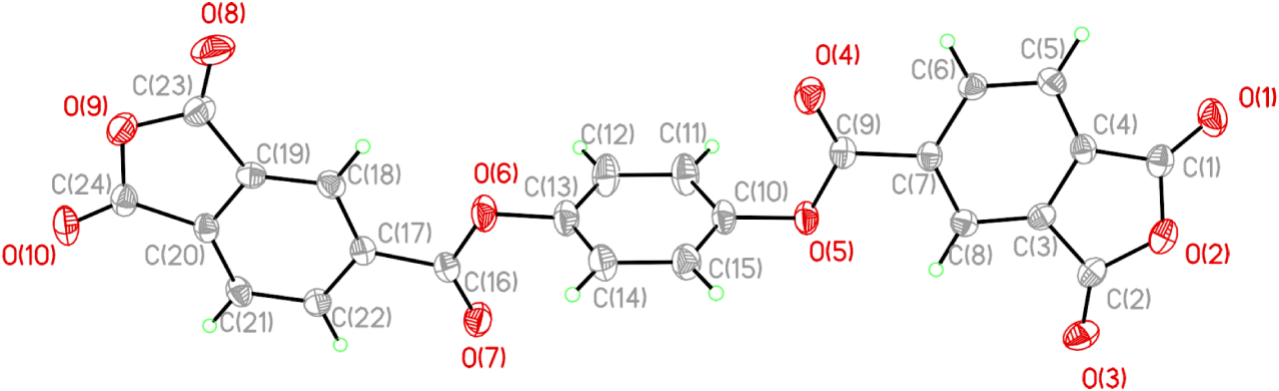
X-ray single crystal structure of TAHQ.

Scheme 1 depicts model trimers based on (a) TAHQ–3,4-ODA–TAHQ and (b) TAHQ–4,4-ODA–TAHQ. Within this qualitative model, the TAHQ–3,4-ODA segment appears to adopt a more extended (less torsionally distorted) path than the 4,4-linked analog under our chosen constraints, suggesting packing motifs that are favorable for reducing dielectric loss.

**Scheme 1:**
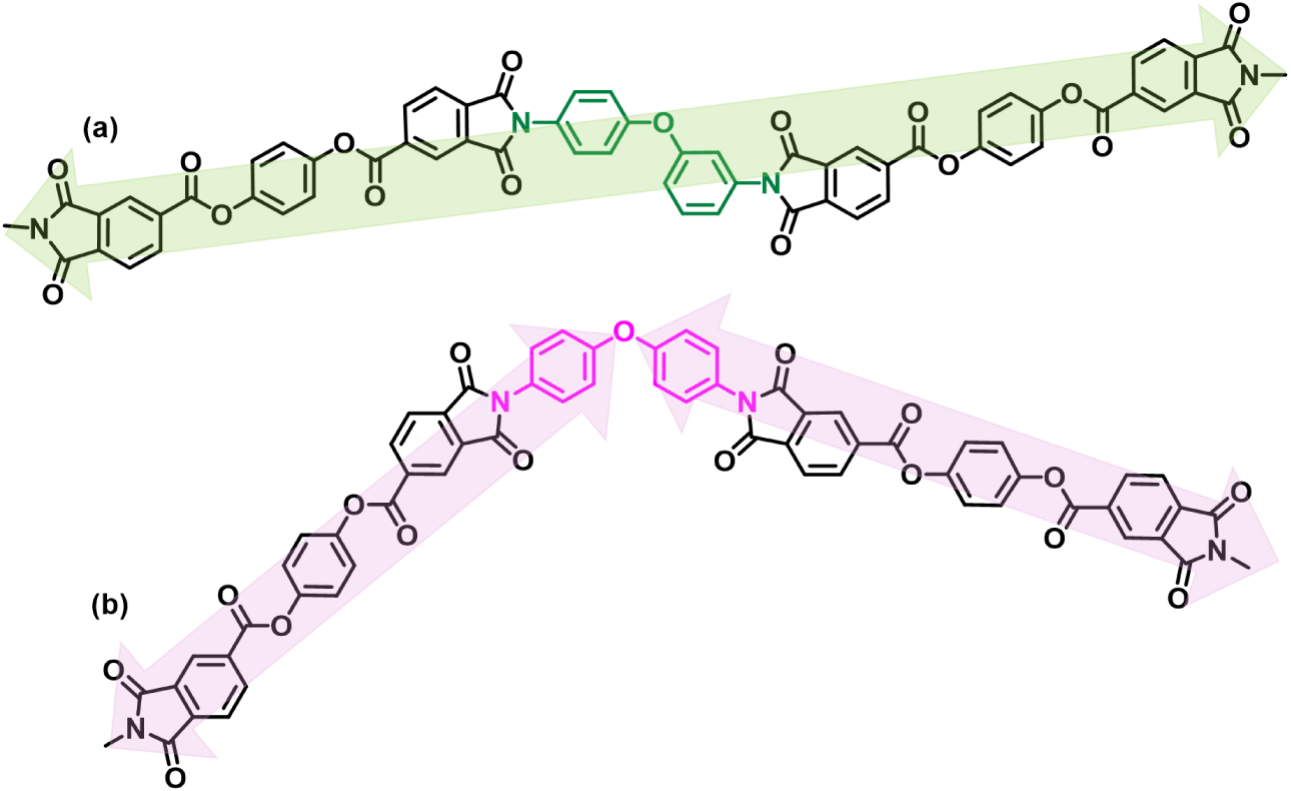
Structures of (a) TAHQ–3,4-ODA–TAHQ and (b) TAHQ–4,4-ODA–TAHQ Trimers

Experimentally, we found that the PI from 3,4-ODA/TAHQ (PI-1) exhibits a lower *D*_f_ than the literature-recommended 4,4-ODA/TAHQ,^35^ indicating that 3,4-ODA is an effective building block for low-*D*_f_ polyimides. A very recent paper also reports that 3,4-ODA-based polyimides show a lower *D*_f_ than 4,4-ODA-based polyimides,^1^ but the CTE is in the range of 35–45 ppm/°C, which is not satisfied for flexible printed circuit boards. To further reduce the loss of the m-tolidine/TAHQ polyimide, we incorporated 3,4-ODA to form PI-X type copolyimides (where X is the mole fraction of 3,4-ODA in the diamine feed; Scheme 2 and Table S3). In Scheme 2, the blue “mesogen biphenyl unit” (from m-tolidine) increases segmental rigidity and promotes in-plane chain alignment during film formation, suppressing high-frequency dipolar relaxation. The green “flexible linear ether unit” (from 3,4-ODA) acts as a nearly linear, conformationally compliant spacer that improves backbone linearity versus 4,4-ODA and enhances processability and reactivity. Notably, ether-based diamines are more reactive toward dianhydrides than ester-based diamines; the latter often display reduced reactivity and, by making the backbone excessively rigid, can compromise toughness and overall mechanical performance. The red “mesogen ester unit” (from the TAHQ ester dianhydride) provides a rigid, planar bridge that preserves the backbone linearity. This simple copolymerization approach, without complex monomer design or multistep diamine syntheses, yields films with *D*_f_ as low as 0.0013 and a minimum CTE of 11.8 ± 2.8 (*n* = 4) ppm/°C while maintaining robust thermal and mechanical properties. Thus, introducing a controlled fraction of 3,4-ODA offers a practical route to lower *D*_f_ values in fluorine-free PIs for high-frequency electronic applications.

**Scheme 2:**
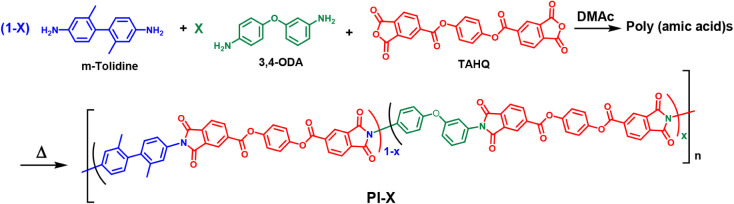
Synthesis of Polyimides (PI-X)

In this study, a series of fluorine-free PI-X copolyimides were prepared by copolymerizing TAHQ with m-tolidine and 3,4-ODA at different diamine feed ratios. The detailed preparation procedures of the copolyimides are described, and their thermal, mechanical, dielectric, optical, and structural properties were systematically investigated to clarify the structure–property relationships responsible for the ultralow dielectric loss and thermal expansion close to that of copper.

## 2 Experimental Section

### 2.1 Materials

1,4-Phenylene bis­(1,3-dioxo-1,3-dihydroisobenzofuran-5-carboxylate) (TAHQ) (>95.0%), 3,4′-oxidianiline (3,4-ODA) (98.0%), 4,4′-oxidianiline (4,4-ODA) (98.0%), 4,4′-diamino-2,2′-dimethylbiphenyl (m-tolidine) (98.0%), 4,4′-oxidiphthalic anhydride (ODPA) (>98.0%) were purchased from TCI. Dimethylacetamide (DMAc, >99.0%) was purchased from Duksan. Acetic anhydride (97.0%) was purchased from ECHO Chemicals. All chemicals are reagent grade, and solvents are of ACS or HPLC grades.

### 2.2 Characterization

Structural and thermal characterizations were conducted by using standard analytical techniques. Nuclear magnetic resonance (NMR) spectra of monomers were acquired on Agilent 400 and 600 MHz spectrometers, with DMSO-*d*_6_ or CDCl_3_ as solvents, depending on solubility. Infrared (IR) spectra were measured by using a PerkinElmer RX1 spectrometer in the 400–4000 cm^–1^ range. Inherent viscosity was determined in DMAc (0.5 g/dL, 30 °C) using PAA isolated by precipitation into excess methanol, followed by filtration and vacuum drying. Thermogravimetric analysis (TGA) was carried out under nitrogen using a PerkinElmer Pyris 1 instrument, heating samples at a rate of 20 °C/min to assess thermal degradation behavior. Dynamic mechanical analysis (DMA) was performed with a PerkinElmer Pyris Diamond system to evaluate viscoelastic properties; temperature-dependent storage modulus (E′) and loss factor (tan δ) were recorded at 1 Hz frequency and a 5 °C/min heating rate. Thermomechanical analysis (TMA) was performed using an SII TMA/SS6100 instrument in expansion mode. Film specimens with dimensions of 0.5 cm × 2.0 cm were heated from 40 to 300 °C at 10 °C/min under a constant static force of 100 mN. Each sample was measured four times, and the reported CTE values represent the average calculated over 50–150 °C. Tensile properties of the PI films were determined at room temperature using a Shimadzu EZ-SX universal tester. Film strips (5 cm × 1 cm) were stretched under a 100 N load at a crosshead speed of 5 mm min^–1^; tensile strength, elongation at break, and Young’s modulus are reported as mean ± standard deviation (*n* = 4). Refractive indices in both the in-plane (*n*_TE_) and out-of-plane (*n*_TM_) directions were measured at 633 nm using a Metricon-2010 prism-coupling refractometer at room temperature. Water absorption (*W*_A_) of the PI-X films was calculated using the equation: *W*_A_ (%) = (*W* – *W*_0_)/*W*_0_ × 100, where *W*_0_ represents the weight of the PI-X film after vacuum drying at 100 °C for 24, and *W* is the weight after immersion in water at room temperature for 24, 48, and 72 h followed by gentle wiping with tissue paper. Wide-angle X-ray diffraction (WXRD) was performed using a Bruker D8 DISCOVER SSS diffractometer equipped with high-power capability, scanning over a 2θ range of 5–50° to evaluate molecular packing and structural order. The dielectric constant (*D*_k_) and dissipation factor (*D*_f_) at 10 GHz under dry conditions were measured in our laboratory using an R&S ZNB vector network analyzer. Circular film samples (approximately 5 cm in diameter) were used for the analysis. Humidity-conditioned dielectric measurements (RH = 0%, 50%, and 100% for 24 h) were performed for selected samples (PI-0.5 and PI-0.625) at an external industrial testing facility using an Agilent E5071C vector network analyzer.

### 2.3 General Synthesis Procedures of Polyimides

All reactions were performed in oven-dried glassware under dry N_2_. DMAc was dried over 4 Å molecular sieves. Before use, dianhydrides were dried at 120 °C (vacuum, ≥8 h) and diamines at 80 °C (vacuum, ≥8 h). In a 100 mL three-neck flask under N_2_, the diamines m-tolidine and 3,4-ODA were dissolved in dry DMAc to give a total diamine feed of 6.5 mmol (m-tolidine = 6.5 (1–X) mmol; 3,4-ODA = 6.5X mmol; X = 0, 0.25, 0.375, 0.50, 0.625, 0.75, or 1). TAHQ (6.5 mmol) was added proportionally at room temperature, and the mixture was stirred for 12 h to afford a poly­(amic acid) (PAA) solution (Table S3). After brief degassing (vacuum/N_2_ sparging), PAA was cast onto clean glass and thermally imidized under N_2_ using the schedule 80 °C/12 h, 100 °C/1 h, 200 °C/1 h, and 300 °C/1 h with 2–3 °C min^–1^ ramps to give copolyimide films denoted PI-X, where X is the mole fraction of 3,4-ODA in the diamines and can be 0, 0.25, 0.375, 0.5, 0.625, 0.75, and 1. Free-standing films were peeled, dried at 110 °C (vacuum, 2 h) before testing. 4,4-ODA/TAHQ and 3,4-ODA/ODPA copolyimides were prepared identically by substituting the corresponding equimolar monomers (4,4-ODA with TAHQ, or 3,4-ODA with ODPA; each 6.5 mmol) and following the same casting and imidization protocol.

## 3 Results and Discussion

### 3.1 Structure Analysis

Owing to their pronounced rigidity and densely compacted molecular structure, thermally imidized polyimide (PI) films exhibit intractable insolubility in standard deuterated solvents. This inherent characteristic consequently precludes their utility for solution-state NMR spectroscopy. Structural confirmation was therefore effected through FTIR spectroscopy (Figure S1). All spectra display the characteristic imide absorptions: the asymmetric C=O stretch at ∼1782 cm^–1^, the symmetric C=O stretch at ∼1725 cm^–1^, the C–N–C stretching band at ∼1383 cm^–1^, and the imide ring deformation near ∼725 cm^–1^. The paired carbonyl bands (∼1782/1725 cm^–1^) reflect the two nonequivalent imide carbonyls and are diagnostic of imide formation; the strong ∼1383 cm^–1^ band arises from C–N–C stretching within the imide linkage, and the ∼725 cm^–1^ band corresponds to the five-membered ring deformation. Concurrently, no signals attributable to amic-acid O–H/N–H or residual dianhydride carbonyls are observed. Collectively, these features indicate complete cyclodehydration and the successful formation of the targeted aromatic PI without detectable side products. Table S3 summarizes the inherent viscosity of the corresponding PAA. The inherent viscosity decreases gradually with increasing 3,4-ODA content. This trend is consistent with the electronic characteristics of 3,4-ODA: the ether linkage is an electron-donating substituent that can increase aromatic amine nucleophilicity via resonance donation primarily to the *ortho*- or *para*-positions, but it cannot effectively resonate with a *meta*-positioned amino group. Consequently, one of the amine groups in 3,4-ODA is less activated, which may reduce the overall effective reactivity and lead to a lower inherent viscosity. Nevertheless, the viscosity remains sufficient for solution casting, and PI-1 still forms a flexible film. The PAA solution also showed good storage stability at 4 °C; for example, the inherent viscosity of PI-0.5 changed only slightly from 1.04 dL/g initially to 1.03 dL/g after 48 h and 1.01 dL/g after 96 h (Table S3). Figure S2 shows the ^1^H NMR spectra of m-tolidine, 3,4-ODA, and 4,4-ODA. The amino proton signals of m-tolidine and 4,4-ODA appear at 4.87 and 4.79 ppm, respectively, whereas those of 3,4-ODA appear at 4.92 and 5.10 ppm. The downfield shift of the amino protons in 3,4-ODA indicates a more electron-deficient environment, suggesting lower nucleophilicity and lower reactivity toward dianhydride. This result supports the observed decrease in PAA viscosity with an increasing 3,4-ODA content. Figure S3 shows the ^1^H NMR spectra of the corresponding PAA in DMSO-*d*_6_. The broad signal at 10.5–11.3 ppm is attributable to the carboxylic acid (−COOH) of the amic acid. Moreover, quantitative integration supports the designed copolymer composition: after normalizing the m-tolidine methyl peak to an integral of 6.00 (two CH_3_ groups), the expected aromatic-proton integral increases with 3,4-ODA content because the 3,4-ODA/TAHQ repeat unit contains two more aromatic protons than the m-tolidine/TAHQ unit (18 vs 16). Accordingly, the predicted aromatic integrals are 16.00 (PI-0), 22.00 (PI-0.25), and 34.00 (PI-0.5) under this normalization, in excellent agreement with the measured values shown in Figure S3.

### 3.2 Visual Appearance of the Polyimide Films

The polyimide films were prepared by solution polycondensation, followed by thermal imidization. As shown in Figure S4, all formulations form flexible, defect-free yellow films with good visible clarity, and the observed color/clarity variations are consistent with a charge-transfer-complex (CTC) mechanism. The PI-0 is the palest, as the m-tolidine unit’s meta linkages and pendant methyl groups disrupt backbone coplanarity and π–π stacking, suppressing CTC formation and thus reducing coloration. As the fraction of the ether-containing diamine 3,4-ODA increases, the films become progressively darker: the ether oxygen donates electron density into the aromatic system, enhancing the donor character of the diamine; paired with an electron-deficient dianhydride, this strengthens donor–acceptor interactions and promotes CTCs, yielding a deeper yellow-brown hue and slightly lower transmittance. The PI-1 is the darkest and least transparent, consistent with its more planar, symmetrical backbone that favors tighter stacking and stronger CTCs. Overall, structures that disrupt planarity/stacking (e.g., m-tolidine) produce lighter films, whereas increased donor strength and backbone planarity (higher 3,4-ODA) intensify CTCs and deepen the color.

### 3.3 Mechanical Properties

Tensile properties of the polyimide films were measured using a universal testing machine in tension mode to determine tensile strength (MPa) and elongation at break (%), while the Young’s modulus (GPa) was derived from the initial slopes of stress–strain curves, and the average results are reported using the standard deviation of 4 replicates (Figure S5). Tensile strength reflects the maximum stress sustained before failure; elongation at break indicates overall deformability or ductility at fracture; and Young’s modulus characterizes stiffness in the elastic regime. Figure 2 summarizes the tensile properties of polyimides PI-X. The tensile strength peaks at 156.8 ± 5.3 MPa for PI-0.25, suggesting optimal chain packing and rigidity at low 3,4-ODA content. As the proportion of 3,4-ODA increases, both tensile strength and modulus gradually decline due to the enhanced segmental flexibility introduced by ether linkages. Meanwhile, elongation at break increases moderately, indicating improved ductility. PI-1, with the highest ether content, shows the lowest tensile strength (68.0 ± 3.5 MPa) and modulus (1.27 ± 0.09 GPa) but retains good elongation (5.2 ± 0.23%) and acceptable mechanical robustness. Overall, polyimides PI-X exhibit tensile strengths ranging from 68 to 157 MPa, sufficient to meet the mechanical requirements for flexible printed circuit board (FPCB) applications.

**Figure 2:**
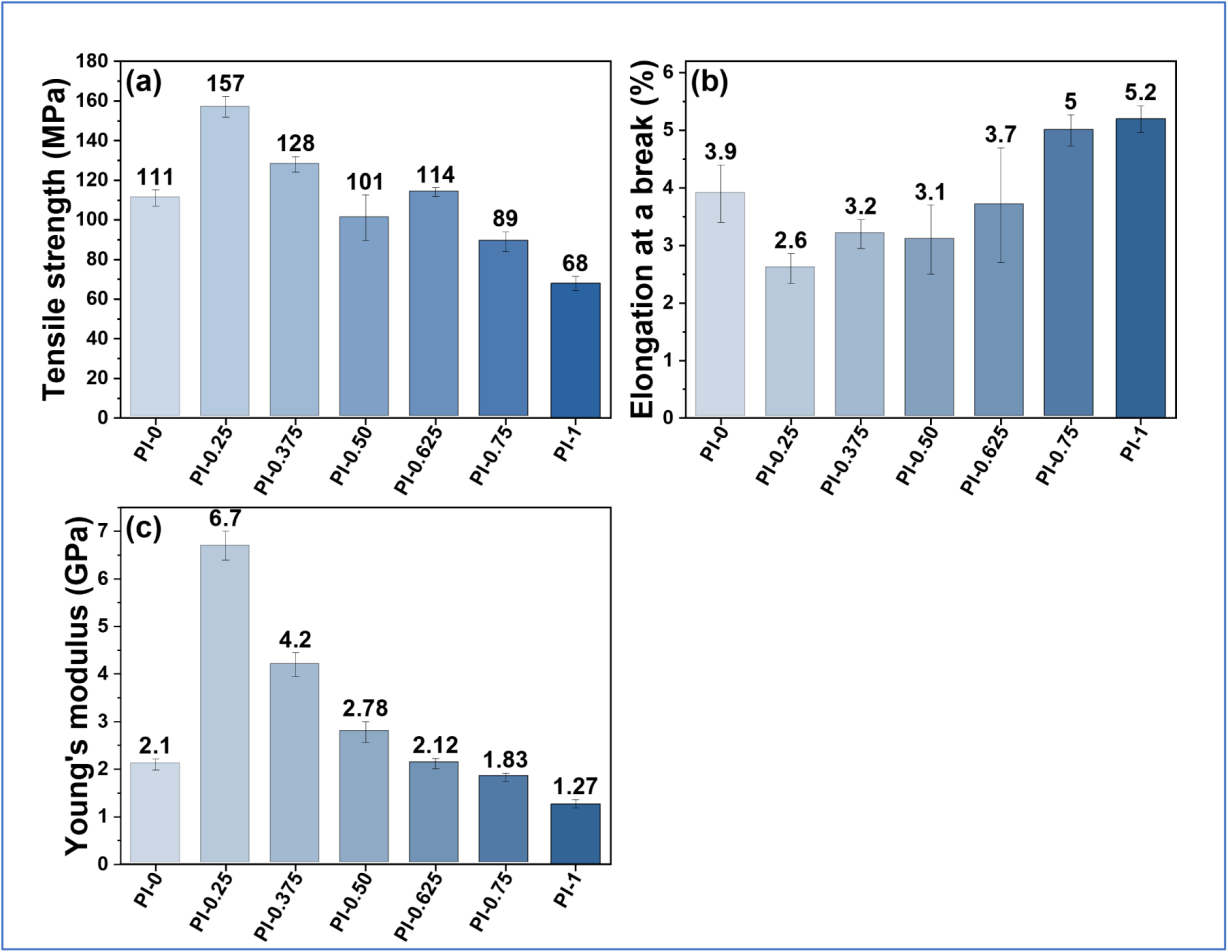
(a) Tensile strength, (b) elongation at break, and (c) Young’s modulus of PI-X films (mean ± SD, *n* = 4).

### 3.4 Thermal Properties

Dynamic mechanical analysis was used to record the storage modulus E′, loss modulus E″, and loss factor tan δ. The glass transition temperature (*T*_g_) was taken from the tan δ peak. As shown in Figure 3a (with data summarized in Table 1), all PI-X films maintain a high storage modulus up to the measurement limit and show no obvious tan δ peak below 350 °C, indicating *T*_g_ (DMA) > 350 °C for the entire series. The persistence of a high E′ and the suppressed relaxation are attributed to the combined effects of the aromatic imide backbone and the ordered arrangement of relatively rigid and linear chain segments, which strengthen intermolecular interactions and restrict segmental motion. A weak shoulder at higher temperatures is attributable to a β-relaxation associated with local rotations near flexible linkages (e.g., ether bridges), consistent with reports for aromatic polyimides.^36–39^ Thermomechanical analysis (TMA) further confirms excellent dimensional stability (Figure 3b). To ensure reliability and reproducibility, TMA thermograms were measured with four independent replicates (Figure S6) and the replicate statistics are in Table S5. The CTE values in Table 1 have been updated accordingly using the average and standard deviation (50–150 °C). All samples exhibit *T*_g_ (TMA) > 300 °C, while the in-plane CTE (50–150 °C) shows a pronounced nonlinear dependence on composition. Specifically, introducing a moderate fraction of 3,4-ODA lowers the CTE to 13.4 ± 3.3 ppm/°C (PI-0.25) and reaches a minimum of 11.8 ± 2.8 ppm/°C (PI-0.375), whereas further increasing 3,4-ODA raises the CTE to 17.2 ± 1.6 ppm/°C (PI-0.625), 21.6 ± 1.4 ppm/°C (PI-0.75), and 31.1 ± 1.9 ppm/°C (PI-1) (Table 1). This nonmonotonic behavior cannot be rationalized by a simple rigidity averaging. Instead, it is consistent with a structure-driven packing: the WXRD patterns (Figure 4) reveal that the PI-X series exhibits superposed crystalline features, unlike the ether-type control (3,4-ODA/ODPA), evidencing the crystallization-promoting effect of the rigid, ester-containing TAHQ scaffold. Notably, compositions with intermediate 3,4-ODA content show enhanced diffraction intensities (Figure 4), implying tighter local stacking and stronger short-/medium-range order. Such ordered packing is expected to constrain in-plane segmental dilation and thereby suppress the in-plane CTE measured by TMA. When the 3,4-ODA fraction becomes too high, additional ether linkages increase conformational freedom, which broadens the crystalline features (Figure 4) and leads to increased thermal expansion. From an application standpoint, PI-0.625 provides a copper-matched CTE (∼17 ppm/°C), which is beneficial for mitigating interfacial stress and warpage in copper-clad laminates. The comprehensive CTE comparison of the PI-X series with various other low-dielectric property PIs is shown in Figure 3e and summarized in Table S6.^40–46^ The PI-X polyimides exhibit coefficients of thermal expansion close to that of copper, highlighting their promise for a wide range of electronic devices, particularly in printed circuit board fabrication. Figure 3c and d shows TGA and DTG thermograms, respectively. Thermal stability was evaluated under nitrogen using the 5% weight-loss temperature *T*_d5%_, char yield at 800 °C and the peak temperature of the DTG curve (*T*_max_). All copolyimides exhibit excellent thermal stability with *T*_d5%_ ∼ 485–500 °C and *T*_max_ = 503–527 °C. Char yields are higher than 42% for m-tolidine-containing copolyimides, whereas the ether-rich PI-1 has the lowest value of 33.0%. The two-stage DTG behavior corresponds first to scission of lower-bond-energy linkages (ether/ester segments and initial imide opening, ∼400–580 °C), followed by degradation/carbonization of the aromatic backbone at higher temperatures (∼600–750 °C). Overall, the fully aromatic, imide-rich architecture confers high *T*_g_, reduced CTE, and robust thermal stability with significant char formation.

**Figure 3:**
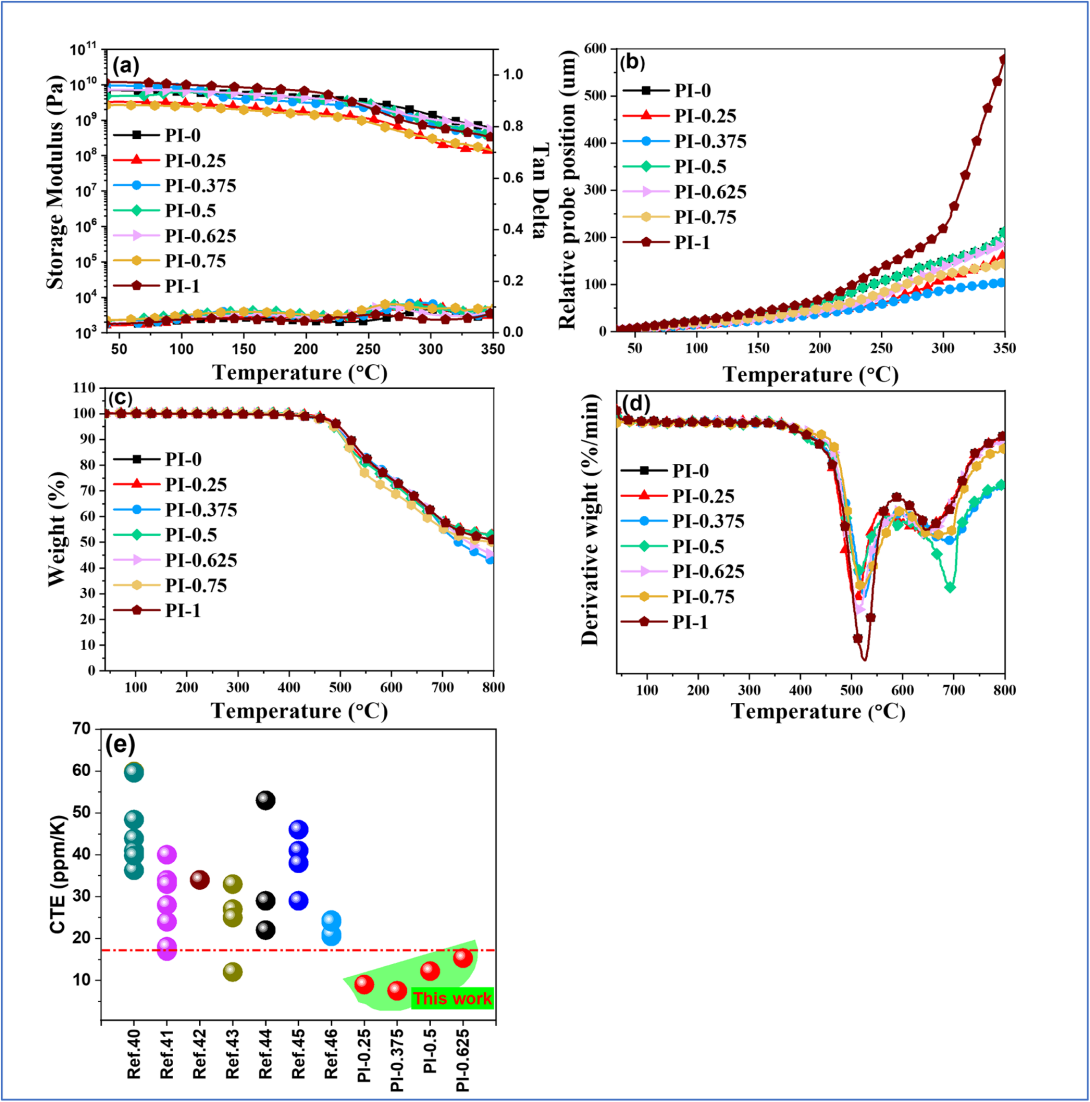
(a) DMA, (b) TMA, (c) TGA, and (d) DTG thermograms of PI-X; and (e) comparison of the in-plane CTE values of the PI-X series with those of various low-dielectric polyimides reported in the literature, including only data measured in the temperature range of 50–150 °C. The CTE of copper (17 ppm/°C) is indicated by a dashed line.

**Table 1:** Thermal Properties of PI-X Series Copolyimides

Sample code	*T*_g_ (DMA) (°C)^a^	*T*_g_ (TMA) (°C)^b^	CTE (ppm/°C)^c^	*T*_d5%_ (°C)^d^	Char yield (%)^e^	*T*_max_ (°C)^f^	*n*_TE_	*n*_TM_	Δ*n*
PI-0	>350	>300	13.7 ± 0.2	487	49.8	524	1.772	1.5966	0.1755
PI-0.25	>350	>300	13.4 ± 3.3	487	52.5	527	1.756	1.5916	0.1639
PI-0.375	>350	>300	11.8 ± 2.8	493	44.1	526	1.748	1.5913	0.1571
PI-0.5	>350	>300	15.6 ± 2.4	490	41.9	503	1.7552	1.6010	0.1542
PI-0.625	>350	>300	17.2 ± 1.6	496	52.5	512	1.769	1.6041	0.1652
PI-0.75	>350	>300	21.6 ± 1.4	498	50.4	513	1.753	1.5964	0.1565
PI-1	>350	>300	31.1 ± 1.9	486	33.0	524	1.742	1.6029	0.139
4,4-ODA/TAHQ							1.708	1.6165	0.0911
3,4-ODA/ODPA							1.683	1.6818	0.0008

a Measured by DMA at a heating rate of 5 °C/min. *T*_g_ was determined from a peak temp of tan δ curve.

b Measured by TMA at a heating rate of 10 °C/min.

c CTE was determined from TMA measurements over the range of 50–150 °C, and the reported values are the mean ± standard deviation from four independent replicates (*n* = 4).

d The initial decomposition temperature (*T*_d5%_) was obtained from TGA analysis at a heating rate of 20 °C/min under a nitrogen atmosphere.

e Measured by TGA at a heating rate of 20 °C/min under a nitrogen atmosphere. Char yield was taken from the TGA curve at 800 °C.

f *T*_max_ was obtained from the DTG curve and represents the temperature corresponding to the maximum decomposition rate.

**Figure 4:**
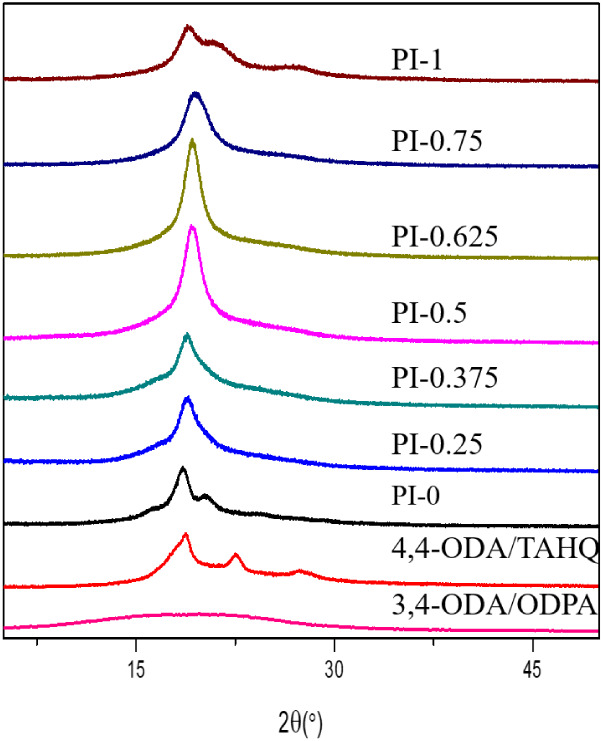
XRD patterns of PI-X, 4,4-ODA/TAHQ, and 3,4-ODA/ODPA.

### 3.5 X-ray Diffraction and Water Absorption

Wide-angle X-ray diffraction (WXRD) was employed to probe chain stacking and short-/medium-range order in the polyimide films, as local aggregation and packing coherence can restrict segmental motion and thereby influence both dimensional stability and dielectric dissipation. Figure 4 shows WXRD of PI-X, along with 4,4-ODA/TAHQ and 3,4-ODA/ODPA. Unlike the ether-type control 3,4-ODA/ODPA displaying only a single amorphous halo, the PI-X series exhibits superposed ordered/crystalline-related features on the amorphous background. This observation highlights the ordering- and crystallization-promoting role of the rigid, planar TAHQ scaffold, which favors aromatic stacking and enhances packing coherence during film formation. Among the PI-X compositions, copolyimides containing a moderate fraction of 3,4-ODA (e.g., PI-0.5 and PI-0.625) present the highest diffraction intensities, suggesting the most coherent local stacking and the highest degree of short-/medium-range order. As the 3,4-ODA content increases, the broad halo shifts slightly toward higher 2θ and the calculated *d*-spacing decreases (Table S4), indicating a reduction in the average interchain distance. Notably, at very high 3,4-ODA loadings, the halo becomes broader and weaker despite the smaller mean spacing, which is consistent with additional ether linkages increasing conformational freedom and disrupting coherent packing (i.e., reduced correlation length) even when chains can approach more closely on average. Overall, the ester-type TAHQ-containing PI-X films preserve short-range order more effectively than the ether-type control, reflecting a stronger mesogenic tendency and higher backbone rigidity. Table S4 summarizes the gravimetric water absorption (*W*_A_, wt %) of the PI-X films after immersion in deionized water for 24, 48, and 72 h. The WA values approach a plateau within 24 h, indicating that the PI-X films rapidly reach near-equilibrium uptake under these conditions. As 3,4-ODA increases, water absorption decreases gradually. The decrease is monotonic up to PI-0.75 (1.52%) and shows a slight rebound at PI-1 (1.61%), but the overall trend clearly indicates reduced hygroscopicity at higher 3,4-ODA content. Although ether linkages are polar, water uptake in aromatic polyimides is not determined solely by bond polarity; chain packing might play a role. In our PI-X series, WXRD shows that increasing 3,4-ODA generally shifts the 2θ peak in the range of 18.5–19.4 toward higher 2θ and reduces the apparent interchain distance, indicating tighter average packing. Notably, PI-0.75 exhibits a smaller apparent interchain distance than PI-1 (4.567 Å vs 4.697 Å), suggesting more compact/coherent local stacking at PI-0.75. Such densified packing is expected to restrict water penetration and reduce accessible free volume, providing a plausible structural explanation for the observed decrease in WA with increasing 3,4-ODA content.

### 3.6 Refractive Index (Birefringence)

The refractive properties of PI-X, 4,4-ODA/TAHQ, and 3,4-ODA/ODPA were characterized to probe the chain arrangement in the films. Table 1 summarizes the refractive indices measured in the TE and TM modes at 633 nm, from which the birefringence (Δ*n* = *n*_TE_ – *n*_TM_) was calculated. With the diamine fixed as 3,4-ODA, PI-1 (3,4-ODA/TAHQ) exhibits a dramatically larger birefringence than the ether-type control 3,4-ODA/ODPA (Δ*n* = 0.139 vs 0.0008; Table 1), indicating much stronger optical anisotropy associated with anisotropic chain arrangement in the film. This pronounced increase in Δ*n* upon replacing ODPA with TAHQ highlights the pivotal role of the dianhydride structure in promoting ordering/orientation. In particular, the rigid and highly symmetric aromatic backbone of TAHQ is expected to enhance intermolecular packing and restrict conformational freedom, thereby favoring anisotropic chain alignment during film formation. This interpretation is consistent with the elongated, rod-like conformation of TAHQ (Figure 1). Within the TAHQ-based system, PI-1 (3,4-ODA/TAHQ) also shows a higher Δn than 4,4-ODA/TAHQ (Δ*n* = 0.139 vs 0.0911; Table 1), suggesting that the diamine geometry is another key determinant of the orientation tendency. The larger birefringence of the 3,4-ODA-derived polyimide implies a greater disparity between the in-plane and out-of-plane refractive indices, consistent with our design rationale that 3,4-ODA is more favorable than 4,4-ODA for promoting molecular ordering/orientation when paired with the rigid TAHQ dianhydride (Scheme 1). Birefringence provides a sensitive indicator of anisotropic chain arrangement that links the film microstructure to macroscopic dimensional stability. In the present system, the pronounced Δ*n* values suggest the development of an anisotropic packing during film formation rather than purely isotropic densification. This interpretation is consistent with the WXRD results (Figure 4), where the PI-X series exhibits ordered/crystalline-related features superposed on the amorphous halo and enhanced diffraction intensity for intermediate compositions, indicating more coherent local stacking. Such anisotropic ordering and coherent packing are expected to constrain in-plane segmental dilation, thereby suppressing the in-plane CTE measured by TMA (Figure 3b). Collectively, the Δ*n*–WXRD–CTE correlations support an “order–flexibility balance” in this copolyimide system: a moderate 3,4-ODA content may provide sufficient conformational adjustability to achieve coherent packing under the TAHQ-driven stacking motif, whereas excessive 3,4-ODA increases local mobility and ultimately raises thermal expansion.

### 3.7 Dielectric Properties

Figure 5a shows the *D*_k_ and *D*_f_ values of PI-X, 3,4-ODA/ODPA, and 4,4-ODA/TAHQ measured by the R&SZNB vector network analyzer at 10 GHz. With the diamine fixed as 3,4-ODA, replacing the ether-type dianhydride (ODPA) with the ester-type, linear TAHQ significantly reduces dielectric loss, decreasing *D*_f_ from 0.0045 for 3,4-ODA/ODPA to 0.0017 for PI-1 (3,4-ODA/TAHQ). This improvement is attributed to TAHQ’s mesogenic linearity and its rigid ester character, which together lower the effective density of imide dipoles and suppress high-frequency relaxation processes. With TAHQ held constant, 3,4-ODA yields a lower dielectric loss than 4,4-ODA (PI-1: *D*_f_ = 0.0017 vs 4,4-ODA/TAHQ: *D*_f_ = 0.0023). As illustrated in Scheme 1, the TAHQ–3,4-ODA–TAHQ trimer shows a more linear backbone conformation than the TAHQ–4,4-ODA–TAHQ trimer, contributing to reduced dielectric dissipation. In the PI-Xs, a moderate fraction of 3,4-ODA results in the lowest dielectric losses, with PI-0.5 showing a *D*_f_ at 0.0013, PI-0.625 at 0.0015, and PI-0.375 at 0.0017. At higher 3,4-ODA content, the increased presence of ether oxygens modestly raises the polarity and leads to a slight increase in *D*_f_. To capture overall transmission loss potential, we use *D*_f_ × √*D*_k_ as an evaluation parameter. Figure 5b lists the *D*_f_ × *√D*_k_ value of various polyimides. The value is smaller than 0.0031 for all 3,4-ODA-containing PI-X (e.g., PI-0.5 = 0.0024; PI-0.625 = 0.0028), whereas 3,4-ODA/ODPA is larger (0.0077), confirming the advantage of pairing an ester-type dianhydride with a judicious ether content. Fundamentally, the synergistic ester-ether architecture—which incorporates TAHQ to mitigate the effective imide dipole density and foster linear, short-range molecular order, alongside a judicious inclusion of 3,4-ODA—culminates in ultralow-loss films. Specifically, the PI-0.5 formulation achieves a *D*_f_ as minimal as 0.0013, rendering it exceptionally well suited for high-speed interconnect applications. Figure 5c–d shows the *D*_k_ and *D*_f_ values of PI-0.5 and PI-0.625 at relative humidity (RH) of 0%, 50%, and 100% for 24 h measured by the Agilent E5071C vector network analyzer at 10 GHz. These measurements were conducted by a collaborating polyimide company in Taiwan. *D*_f_ increases monotonically for both compositions, while *D*_k_ remains essentially invariant within experimental scatter (PI-0.5 ≈ 3.2 across RH; PI-0.625 ≈ 3.4–3.5). The pronounced *D*_f_ rise with moisture is consistent with additional dipolar relaxations introduced by absorbed water, whereas the small, nonsystematic *D*_k_ changes suggest minimal net polarization change at these uptake levels. Operationally, this indicates that signal attenuation (via *D*_f_) is the moisture-sensitive parameter, whereas impedance matching (via *D*_k_) is robust in humid environments.

**Figure 5:**
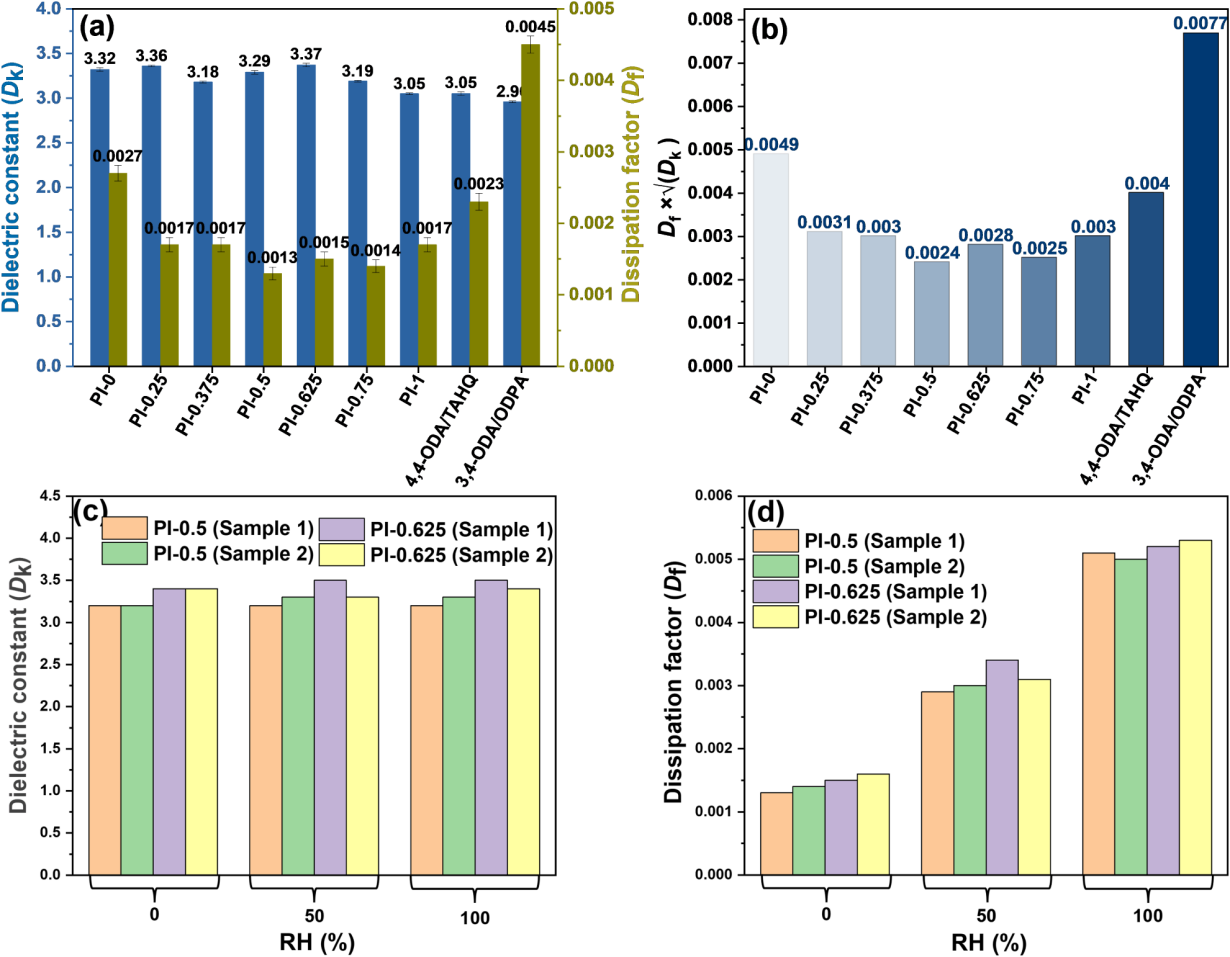
(a) *D*_k_ and *D*_f_ values and (b) *D*_f_ × *√D*_k_ values of PI-X, 4,4-ODA/TAHQ, and 3,4-ODA/ODPA measured at 10 GHz in our laboratory using an R&S ZNB vector network analyzer (dry condition). (c) *D*_k_ and (d) *D*_f_ values of PI-0.5 and PI-0.625 after conditioning at relative humidity (RH) of 0%, 50%, and 100% for 24 h, measured at 10 GHz by using an Agilent E5071C vector network analyzer at an external industrial testing facility.

Figure 6 and Tables S7 show a comprehensive comparison of (a) *D*_f_ and (b) *D*_f_ × √*D*_k_ values of this work (PI-0.5, PI-0.625, and PI-0.75) with various PIs at 10 GHz.^1,2,8,16,27,35,40,47–59^ Although the *D*_k_ values of our PI-X series are generally higher than those of many fluorine-containing polyimides, their value of *D*_f_ × √*D*_k_ (proportional to transmission loss *L*) is superior because *D*_f_ is exceptionally lower compared to other polyimides. This trend is consistent with molecular design: ester-type polyimides typically show slightly higher *D*_k_, while fluorinated polyimides often lower *D*_k_ via bulky substituents that increase the free volume; however, those same features frequently raise *D*_f_ through enhanced segmental mobility or interfacial relaxations. In contrast, our PI-X copolyimides pair moderate *D*_k_ with ultralow *D*_f_, yielding *D*_f_ × √*D*_k_ values that match or outperform fluorinated benchmarks despite the absence of PFAS building blocks.

**Figure 6:**
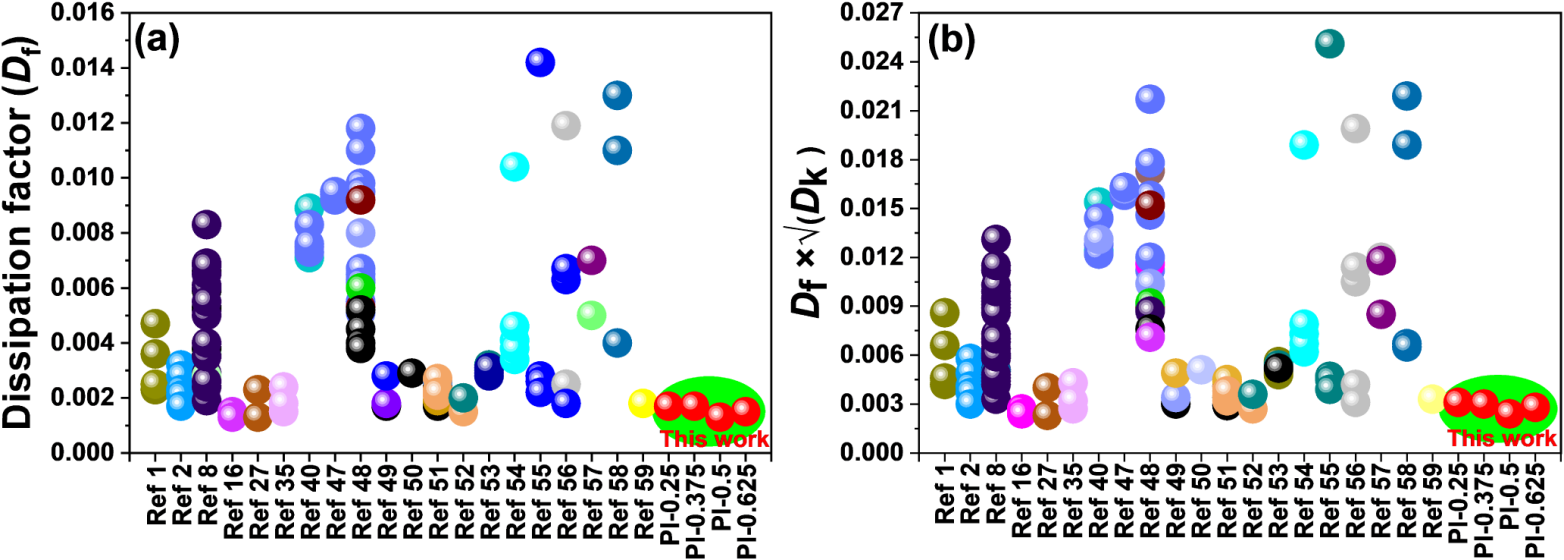
Comprehensive comparison of (a) dissipation factor (*D*_f_) and (b) *D*_f_ × √*D*_k_ of this work with various low-dielectric polyimides.

## 4 Conclusions and Outlook

We established a simple, scalable route to ultralow-loss, fluorine-free polyimides by combining TAHQ with m-tolidine and 3,4-ODA. Across the PI-X series, *D*_f_ reaches as low as 0.0013 at 10 GHz while maintaining *D*_k_ around 3.1–3.4, giving *D*_f_ × √*D*_k_ values of 0.0024–0.0030, which is substantially below the ∼0.004 benchmark for 4,4-ODA/TAHQ and underscoring the excellent low-loss performance of these copolyimides. A composition window of ∼50–62.5 mol % 3,4-ODA affords the best balance of chain orientation and short-range order, suppressing dielectric relaxation without markedly increasing polarity and delivering both lower *D*_f_ and better dimensional control than the corresponding homopolyimides. The PI-X series also exhibit reduced in-plane coefficients of thermal expansion (CTE). The minimum CTE reaches 11.8 ± 2.8 ppm/°C (*n* = 4), while PI-0.625 shows a CTE of 17.2 ± 1.6 ppm/°C (*n* = 4), which closely matches that of copper (∼17 ppm/°C). This close CTE match is beneficial for mitigating interfacial stress, warpage, and reliability issues in copper-clad laminates and related devices. Thermal and mechanical robustness are retained (*T*_d5%_ = 486–498 °C; tensile strength 68–157 MPa), confirming that the cooperative ester–ether design effectively suppresses polarization loss while preserving processability and structural stability. Collectively, these results demonstrate a practical platform for next-generation low-loss polymer dielectrics that simultaneously deliver ultralow *D*_f_ and copper-matched CTE for high-speed and high-frequency interconnects. It should be noted that although 3,4-ODA can promote a more extended effective backbone, the aryl–O–aryl linkage still allows conformational rotation; therefore, the polymer chain is not absolutely linear. To further bias the conformational distribution toward the extended population, we have designed an *ortho*-methyl-substituted 3,4-ODA that sterically suppresses folded/gauche conformations. This strategy provides an additional handle to strengthen chain linearity/packing and to further reduce dielectric loss; the detailed conformational analysis and dielectric structure–property correlation of 3,4-MODA-based copolyimides will be reported separately.

## Supplementary Material



## Data Availability

Access to the data sets substantiating the results presented herein can be obtained from the corresponding author following a suitable inquiry.

## Notes

A complementary (or freely accessible) electronic Supporting Information file is provided, which encompasses a comprehensive array of data. This material includes tabular presentations detailing the crystal data and structural refinement parameters for TAHQ, along with the atomic coordinates and equivalent isotropic displacement parameters. Furthermore, it delineates the synthetic protocol for the PI-X series copolyimides, presents the X-ray Diffraction (XRD) results for the PI films, and furnishes the dielectric characteristics of the PI-X series copolyimides for TAHQ. Visual information is also incorporated, featuring figures illustrating the Fourier-Transform Infrared (FTIR) spectra of PI-0.5, the physical appearance of the PI films, and the mechanical behavior of PI-X. (PDF)
