# Supplementary material for: Facile Strategy Enabling Fluorine-Free Polyimides with Ultralow Dielectric Loss and Thermal Expansion Close to Copper
Source: ACS Appl Mater Interfaces. 2026 Apr 17;18(16):23636–47. doi: 10.1021/acsami.6c02417 (PMC13133785; doi:10.1021/acsami.6c02417)
Supplement: Supplementary file 1 [file am6c02417_si_001.pdf]

Supporting Information

Facile Strategy Enabling Fluorine-Free Polyimides with Ultralow Dielectric Loss and Thermal Expansion Close to Copper

Yu Hsin Liu, Dula Daksa Ejeta, Yi Hsin Chang, Wan Ling Hsiao, Kamani Sudhir K. Reddy, Ching Hsuan Lin*

Department of Chemical Engineering, National Chung Hsing University, Taichung, 40227, Taiwan.

Corresponding Email: linch@nchu.edu.tw

**S. No.**	**Table of contents**	**Page**
1.	Table S1. Crystal data and structure refinement for TAHQ.	3
2.	Table S2. Atomic coordinates (x 10^4) and equivalent isotropic displacement parameters ($Å^2$ x 10^3) for TAHQ. U(eq) is defined as one third of the trace of the orthogonalized U^{ij} tensor.	5
3.	Table S3. Formulation table for the synthesis of PI-X series copolyimides and their inherent viscosities	7
4.	Table S4. XRD and water absorption results of polyimide films.	8
5.	Table S5. Iterative empirical assessments regarding the coefficients of thermal expansion (CTE) for the PI-X.	9
6.	Table S6. Comparison of the in-plane CTE values of the PI-X series with those of various low-dielectric polyimides reported in the literature, including only data measured in the temperature range of 50-150 °C.	9

7. Table S7. Comparison of dielectric properties of this work with various low-dielectric polyimides. 12

8. Figure S1. FTIR spectrum of PI-0.5. 17

9. Figure S2. 18

10. Figure S3. ^{1}H NMR spectra (DMSO-d_6) of PAA precursors for PI-0, PI-0.25, and PI-0.5. Integrals were normalized by setting the m-tolidine methyl peak (two CH_3 groups) to 6.00; the resulting aromatic integrals (16.00, 22.00, 34.00) agree with the theoretical values based on feed composition. Accordingly, the theoretical Ar–H-to-methyl ratios are: 16:6 for PI-0, and (16 x 3/4+18 x 1/4):6 x 3/4=22:6 for PI-0.25, and (16 x 1/2+18 x 1/2):6 x 1/2=34:6 for PI-0.5. These calculated ratios are consistent with the experimental integration results, as shown in Figure S2. 19

11. Figure S4. Visual appearance of polyimide films. 20

12. Figure S5. Stress-strain curves of (a) PI-0, (b) PI-0.5, (c) PI-0.625, and (d) PI-0.75 (each sample was measured 4 times). 21

13. Figure S6. TMA thermograms of (a) PI-0, (b) PI-0.25, (c) PI-0.375, and (d) PI-0.5, (e) PI-0.625, (f) PI-0.75, and (g) PI-1 (each sample measured 4 times). 22

Table S1. Crystal data and structure refinement for TAHQ.

Identification code	TAHQ	
Empirical formula	C24 H10 O10	
Formula weight	458.32	
Temperature	150(2) K	
Wavelength	0.71073 Å	
Crystal system	Orthorhombic	
Space group	$Pna2_1$	
Unit cell dimensions	a = 11.1111(10) Å	a= 90°.
	b = 6.0579(5) Å	b= 90°.
	c = 29.157(3) Å	g = 90°.
Volume	1962.6(3) $Å^3$	
Z	4	
Density (calculated)	1.551 Mg/m^3	
Absorption coefficient	0.124 mm^{-1}	
F(000)	936	
Crystal size	0.430 x 0.200 x 0.070 mm^3	
Theta range for data collection	3.435 to 28.508°.	
Index ranges	-14<=h<=14, -7<=k<=7, -38<=l<=38	
Reflections collected	26018	
Independent reflections	4663 [R(int) = 0.0435]	
Completeness to theta = 25.242°	98.8 %	
Absorption correction	Semi-empirical from equivalents	
Max. and min. transmission	0.9281 and 0.8607	

Refinement method	Full-matrix least-squares on F^2
Data / restraints / parameters	4663 / 1 / 307
Goodness-of-fit on F^2	1.011
Final R indices [I>2sigma(I)]	R1 = 0.0311, wR2 = 0.0840
R indices (all data)	R1 = 0.0344, wR2 = 0.0858
Absolute structure parameter	0.5(2)
Extinction coefficient	n/a
Largest diff. peak and hole	0.215 and -0.152 e.Å^{-3}

Table S2. Atomic coordinates (x 10^4) and equivalent isotropic displacement parameters ($Å^2$ x 10^3) for TAHQ. U(eq) is defined as one third of the trace of the orthogonalized U^{ij} tensor.

	x	y	z	U(eq)
O(1)	3620(2)	16221(3)	2578(1)	39(1)
O(2)	4068(1)	18331(3)	1964(1)	31(1)
O(3)	4807(2)	19487(3)	1282(1)	35(1)
O(4)	8137(1)	10107(3)	1321(1)	36(1)
O(5)	7829(1)	12724(3)	785(1)	28(1)
O(6)	10595(2)	7872(3)	-433(1)	33(1)
O(7)	10111(2)	9969(3)	-1046(1)	34(1)
O(8)	13258(2)	604(3)	-710(1)	49(1)
O(9)	13770(1)	741(3)	-1458(1)	33(1)
O(10)	13945(1)	1755(3)	-2196(1)	38(1)
C(1)	4224(2)	16500(4)	2246(1)	27(1)
C(2)	4821(2)	18146(3)	1578(1)	26(1)
C(3)	5545(2)	16104(3)	1640(1)	22(1)
C(4)	5190(2)	15135(3)	2046(1)	23(1)
C(5)	5671(2)	13163(3)	2199(1)	26(1)
C(6)	6529(2)	12183(3)	1918(1)	24(1)
C(7)	6861(2)	13135(3)	1500(1)	22(1)
C(8)	6388(2)	15146(3)	1354(1)	22(1)
C(9)	7688(2)	11818(3)	1203(1)	24(1)
C(10)	8539(2)	11494(4)	473(1)	27(1)
C(11)	8054(2)	9617(5)	280(1)	37(1)

C(12)	8743(2)	8436(4)	-35(1)	39(1)
C(13)	9878(2)	9184(4)	-140(1)	30(1)
C(14)	10367(2)	11055(4)	54(1)	33(1)
C(15)	9673(2)	12239(4)	371(1)	33(1)
C(16)	10607(2)	8396(4)	-885(1)	25(1)
C(17)	11334(2)	6754(3)	-1148(1)	22(1)
C(18)	11832(2)	4924(4)	-933(1)	25(1)
C(19)	12527(2)	3544(3)	-1200(1)	24(1)
C(20)	12717(2)	3906(3)	-1662(1)	23(1)
C(21)	12198(2)	5692(4)	-1883(1)	27(1)
C(22)	11506(2)	7126(4)	-1619(1)	25(1)
C(23)	13187(2)	1543(4)	-1067(1)	32(1)
C(24)	13522(2)	2132(4)	-1831(1)	27(1)

Table S3. Formulation table for the synthesis of PI-X series copolyimides and their inherent viscosities

Sample code	Diamine		Solvent	Inherent viscosity of PAA (dL/g)[a]
	m-tolidine	3,4-ODA	Dry DMAc	
PI-0	1.390 g (6.5 mmol*1)	0 g	17.6 g	1.32
PI-0.25	1.042 g (6.5 mmol *0.75)	0.328 g (6.5 mmol *0.25)	17.5 g	1.22
PI-0.375	0.868 g (6.5 mmol *0.625)	0.491 g (6.5 mmol *0.375)	17.4 g	1.11
PI-0.5	0.695 g (6.5 mmol *0.5)	0.655 g (6.5 mmol *0.5)	17.4 g	1.04 (1.03[b], 1.01[c])
PI-0.625	0.521 g (6.5 mmol *0.375)	0.819 g (6.5 mmol *0.625)	17.4 g	0.99
PI-0.75	0.347 g (6.5 mmol *0.25)	0.983 g (6.5 mmol *0.75)	17.3 g	0.95
PI-1	0 g	1.311 g (6.5 mmol *1)	17.2 g	0.89

[a] Measured in DMAc at 30 °C at a concentration of 0.5 g/dL (PAA isolated by methanol precipitation and vacuum-dried prior to measurement).
[b] The inherent viscosity after storage in a 4 °C refrigerator for 48 h.
[c] The inherent viscosity after storage in a 4 °C refrigerator for 96 h.

Table S4. XRD and water absorption results of polyimide films.

Sample code	2θ (°)[a]	d (Å)[b]	W_A[c]		
			(24 h)	48 h	72 h
PI-0	18.52 (22.0)	4.787 (4.04)	3.33	3.35	3.33
PI-0.25	18.96	4.677	2.88	2.86	2.88
PI-0.375	18.88	4.697	2.56	2.56	2.56
PI-0.5	19.20	4.619	2.30	2.34	2.31
PI-0.625	19.26	4.605	1.66	1.66	1.66
PI-0.75	19.42	4.567	1.52	1.54	1.52
PI-1	18.88 (22.0)	4.697 (4.04)	1.61	1.62	1.61

[a] For PI-0 and PI-1, the amorphous halo is bimodal; the major peak position is reported in the table, and the second peak at 2θ = 22° (d = 4.04 Å) is reported in parentheses.
[b] Calculated based on Bragg's Law (λ = 1.5406 Å).
[c] W_A: water absorption, wt%.

Table S5. Iterative empirical assessments regarding the coefficients of thermal expansion (CTE) for the PI-X.

Sample ID	CTE (ppm/°C)				Average
	Expt-1	Expt-2	Expt-3	Expt-4	
PI-0	13.5	13.5	13.8	13.8	13.7 ± 0.2
PI-0.25	9.0	11.3	16.2	16.9	13.4 ± 3.3
PI-0.375	7.5	11.1	13.8	13.8	11.8 ± 2.8
PI-0.5	12.2	18.8	16.7	14.6	15.6 ± 2.4
PI-0.625	15.3	19.8	17.1	16.7	17.2 ± 1.6
PI-0.75	19.8	22.1	20.8	23.7	21.6 ± 1.4
PI-1	33.1	32.7	28.2	30.3	31.1 ± 1.9

Table S6. Comparison of the in-plane CTE values of the PI-X series with those of various low-dielectric polyimides reported in the literature, including only data measured in the temperature range of 50-150 °C.

**PEI**	**CTE (ppm/°C)**	**Temperature range (°C)**	**Ref.**

PI-1	59.9	50–150	Main text ref 40
PI-2	59.6	50–150	Main text ref 40
PI-3	43.9	50–150	Main text ref 40
PI-4	41.0	50–150	Main text ref 40
PI-5	48.4	50–150	Main text ref 40
PI-6	36.3	50–150	Main text ref 40
PI-7	39.8	50–150	Main text ref 40
PAI-1a	17	50–150	Main text ref 41
PAI-1b	18	50–150	Main text ref 41
PAI-1c	24	50–150	Main text ref 41
PAI-2a	34	50–150	Main text ref 41
PAI-2b	28	50–150	Main text ref 41
PAI-2c	33	50–150	Main text ref 41
PAI-3a	40	50–150	Main text ref 41
Kapton PI	34	50–150	Main text ref 42
PI/DBCOD-NH_2-0	33	50–150	Main text ref 43
PI/DBCOD-NH_2-0.25	27	50–150	Main text ref 43
PI/DBCOD-NH_2-0.5	25	50–150	Main text ref 43
PI/DBCOD-NH_2-1	12	50–150	Main text ref 43
PEI-5	22	50–150	Main text ref 44
PEI-5’	29	50–150	Main text ref 44
PEI-5’’	53	50–150	Main text ref 44
PSPI-1	46	50–150	Main text ref 45
PSPI-2	41	50–150	Main text ref 45
PSPI-3	38	50–150	Main text ref 45
PSPI-4	29	50–150	Main text ref 45

PSPI-5	29	50–150	Main text ref 45
PI-1	20.5	50–150	Main text ref 46
PI-2	21	50–150	Main text ref 46
PI-3	24.4	50–150	Main text ref 46
PI-4	24	50–150	Main text ref 46
PI-0.25	9.0	50-150	**This work**
PI-0.375	7.5	50-150	**This work**
PI-0.5	12.2	50-150	**This work**
PI-0.625	15.3	50-150	**This work**

Table S7. Comparison of dielectric properties of this work with various low-dielectric polyimides.

PEI	Frequency (GHz)	D_k	D_f	$D_f \times \sqrt{D_k}$	Ref.
TPDA-ODA	10	3.37	0.0047	0.0086	Main text ref 1
TPDA-aODA	10	3.35	0.0023	0.0042	Main text ref 1
TPDA-TPE-Q	10	3.41	0.0036	0.0066	Main text ref 1
TPDA-TPE-R	10	3.42	0.0025	0.0046	Main text ref 1
PEI-NPDA-0	10	3.19	0.0032	0.0058	Main text ref 2
PEI-NPDA-20	10	3.12	0.0028	0.0049	Main text ref 2
PEI-NPDA-50	10	3.07	0.0023	0.0041	Main text ref 2
PEI-NPDA-80	10	3.04	0.0021	0.0037	Main text ref 2
PEI-NPDA-100	10	2.90	0.0017	0.0030	Main text ref 2
IBFA-ABHQ	10	3.02	0.0035	0.0061	Main text ref 8
IBFA-BPTP	10	3.15	0.0029	0.0051	Main text ref 8
IBFA-APAB	10	3.24	0.0038	0.0068	Main text ref 8
IBFA-BAEHF	10	2.90	0.0052	0.0089	Main text ref 8
TAHQ-BAEHF	10	2.67	0.0036	0.0059	Main text ref 8
TAHQ-ABHQ	10	3.03	0.0019	0.0033	Main text ref 8
TAHQ-BAPHF	10	2.44	0.0028	0.0044	Main text ref 8
TAHQ-APAB	10	3.32	0.0040	0.0073	Main text ref 8
TAHQ-BPTP	10	3.05	0.0024	0.0042	Main text ref 8
TAHQ-6FPDA	10	2.71	0.0035	0.0058	Main text ref 8
6FDA-APAB	10	2.63	0.0069	0.0112	Main text ref 8
6FDA-ABHQ	10	2.84	0.0061	0.0103	Main text ref 8

6FDA-BAEHF	10	2.49	0.0083	0.0131	Main text ref 8
6FDA-BPTP	10	2.95	0.0053	0.0091	Main text ref 8
6FEDSA-APAB	10	2.48	0.0059	0.0093	Main text ref 8
6FEDSA-ABHQ	10	2.77	0.0029	0.0048	Main text ref 8
6FEDSA-BPTP	10	2.92	0.0027	0.0046	Main text ref 8
6FEDSA-BAEHF	10	2.44	0.0066	0.0103	Main text ref 8
6FEDSA-6FPDA	10	2.53	0.0060	0.0095	Main text ref 8
6FEDSA-BAPHF	10	2.94	0.0050	0.0086	Main text ref 8
DATFMB-APAB	10	3.01	0.0037	0.0064	Main text ref 8
DATFMB-ABHQ	10	3.16	0.0028	0.0050	Main text ref 8
DATFMB-BPTP	10	3.46	0.0026	0.0048	Main text ref 8
DATFMB-BAEHF	10	3.26	0.0055	0.0099	Main text ref 8
DATFMB-6FPDA	10	2.53	0.0065	0.0103	Main text ref 8
DATFMB-BAPHF	10	2.81	0.0035	0.0059	Main text ref 8
BPADA-APAB	10	3.18	0.0038	0.0068	Main text ref 8
BPADA-ABHQ	10	2.98	0.0035	0.0060	Main text ref 8
BPADA-BPTP	10	3.09	0.0040	0.0070	Main text ref 8
BPADA-BAEHF	10	2.88	0.0068	0.0115	Main text ref 8
BPTP-ODPA	10	3.1954	0.0015	0.0027	Main text ref 16
BPTP-TAHQ	10	3.3234	0.0013	0.0024	Main text ref 16
A3EB-6FDA	10	3.00	0.0023	0.0040	Main text ref 27
A1E-TA2EB	10	3.24	0.0013	0.0023	Main text ref 27
A2EB-TA2EB	10	3.26	0.0013	0.0023	Main text ref 27
A3EB-TA2EB	10	3.29	0.0013	0.0023	Main text ref 27
A1E-ODPA	10	3.24	0.0024	0.0043	Main text ref 35
A2EB-ODPA	10	3.27	0.0015	0.0027	Main text ref 35

ODA-TA2EB	10	3.26	0.0018	0.0032	Main text ref 35
PI-1	10	2.99	0.0089	0.0154	Main text ref 40
PI-2	10	3.05	0.0071	0.0124	Main text ref 40
PI-3	10	2.96	0.0071	0.0122	Main text ref 40
PI-4	10	2.93	0.0075	0.0128	Main text ref 40
PI-5	10	2.79	0.0073	0.0122	Main text ref 40
PI-6	10	3.03	0.0083	0.0144	Main text ref 40
PI-7	10	2.98	0.0076	0.0131	Main text ref 40
ISS/TFDB	10	2.96	0.0092	0.0158	Main text ref 47
CoPI (ISS9-ISM1)	10	2.96	0.0094	0.0162	Main text ref 47
CoPI (ISS7-ISM3)	10	2.92	0.0095	0.0163	Main text ref 47
BPDAODA	10	3.35	0.0080	0.0146	Main text ref 48
BPDABAPP	10	3.17	0.0063	0.0112	Main text ref 48
PMDABAPP	10	3.12	0.0098	0.0173	Main text ref 48
ODPAODA	10	3.37	0.0118	0.0217	Main text ref 48
ODPABAPP	10	3.20	0.0065	0.0116	Main text ref 48
BPADAODA	10	3.19	0.0067	0.0120	Main text ref 48
BPADABAPP	10	3.16	0.0051	0.0091	Main text ref 48
PMDA-6FPDA	10	2.61	0.0110	0.0178	Main text ref 48
PMDABAPHF	10	2.79	0.0062	0.0104	Main text ref 48
ODPA-6FPDA	10	2.75	0.0095	0.0158	Main text ref 48
ODPA-BAPHF	10	2.81	0.0055	0.0092	Main text ref 48
BPADA-6FPDA	10	2.83	0.0053	0.0089	Main text ref 48
BPADA-BAPHF	10	2.85	0.0042	0.0071	Main text ref 48
6FDA-ODA	10	2.74	0.0092	0.0152	Main text ref 48
6FDA-6FPDA	10	2.37	0.0060	0.0092	Main text ref 48

6FDA-BAPP	10	2.82	0.0052	0.0087	Main text ref 48
6FDA-BAPHF	10	2.50	0.0045	0.0071	Main text ref 48
TAHQ/APAB	10	3.64	0.0040	0.0076	Main text ref 48
TAHQ/ODA	10	3.46	0.0038	0.0071	Main text ref 48
BAHQ-TFMB	10	3.24	0.0017	0.0030	Main text ref 49
TAHQ-TFMB	10	3.43	0.0018	0.0034	Main text ref 49
NAHQ-TFMB	10	3.12	0.0028	0.0049	Main text ref 49
PI-mmm-T	10	3.2	0.0029	0.0051	Main text ref 50
TAHQ−ABHQ	10	3.12	0.0019	0.0034	Main text ref 51
TAHQ/ABHQ+M20	10	2.77	0.0018	0.0030	Main text ref 51
AHQ/ABHQ+M10	10	2.9	0.0017	0.0029	Main text ref 51
TAHQ/ABHQ+C20	10	2.97	0.0024	0.0041	Main text ref 51
AHQ/ABHQ+C10	10	3.06	0.0022	0.0038	Main text ref 51
TAHQ/ABHQ+B20	10	2.94	0.0022	0.0038	Main text ref 51
AHQ/ABHQ+B10	10	3	0.0022	0.0038	Main text ref 51
TAHQ/ABHQ+N20	10	2.84	0.0026	0.0044	Main text ref 51
AHQ/ABHQ+N10	10	3.04	0.0023	0.0040	Main text ref 51
TAHQ/ABHQ+T20	10	2.81	0.0027	0.0045	Main text ref 51
TAHQ/ABHQ+T10	10	2.88	0.0019	0.0032	Main text ref 51
TAHQ/ABHQ+r-T20	10	2.85	0.0021	0.0035	Main text ref 51
TAHQ/ABHQ+r-T10	10	2.92	0.0022	0.0038	Main text ref 51
A2EB-ODA-TA2EN	10	3.24	0.0015	0.0027	Main text ref 52
A2EB-ODA-TA2EB	10	3.30	0.0020	0.0036	Main text ref 52
PI-Ref	10	3.2	0.0028	0.0050	Main text ref 53
PI-PM	10	3.02	0.0032	0.0056	Main text ref 53

PI-PA	10	3.18	0.0030	0.0053	Main text ref 53
PI-PB	10	3.06	0.0031	0.0054	Main text ref 53
PI-POB	10	2.93	0.0028	0.0048	Main text ref 53
PI-PEB	10	2.94	0.0029	0.0050	Main text ref 53
PI-TEB	10	2.86	0.0031	0.0052	Main text ref 53
PI-B6 (BPDA-DA6)	10	3.34	0.0034	0.0062	Main text ref 54
PI-B12 (BPDA-DA12)	10	3.21	0.0041	0.0073	Main text ref 54
PI-T6 (TAHQ-DA6)	10	3.1	0.0038	0.0067	Main text ref 54
PI-T12 (TAHQ-DA12)	10	2.96	0.0046	0.0079	Main text ref 54
PMDA-ODA	10	3.31	0.0104	0.0189	Main text ref 54
TAHQ/DDSQ-PEI	10	2.81	0.0028	0.0047	Main text ref 55
TAHQ/ODA-PEI	10	3.05	0.0026	0.0045	Main text ref 55
TAHQ/ABHQ-PEI	10	3.08	0.0022	0.0039	Main text ref 55
PMDA/ODA-PI	10	3.12	0.0142	0.0251	Main text ref 55
PI-TAHN	10	2.84	0.0025	0.0042	Main text ref 56
PI-PMDA	10	2.77	0.0063	0.0105	Main text ref 56
PI-5NTDA	10	2.90	0.0067	0.0114	Main text ref 56
PI-6NTDA	10	2.80	0.0119	0.0199	Main text ref 56
PI-TAHQ	10	2.94	0.0018	0.0031	Main text ref 56
PI(ISBA-CBDA)	10	2.86	0.0050	0.0085	Main text ref 57
PI(ISBA-PMDA)	10	2.93	0.0070	0.0120	Main text ref 57
PI(ISBA-BPDA)	10	2.84	0.0070	0.0118	Main text ref 57
PI(ISBA-C95B5)	10	2.88	0.0050	0.0085	Main text ref 57
PI-1	10	2.84	0.0040	0.0067	Main text ref 58
PI-2	10	2.95	0.0110	0.0189	Main text ref 58
PI-3	10	2.83	0.0040	0.0067	Main text ref 58

PI-4	10	2.83	0.0130	0.0219	Main text ref 58
PI-5	10	2.63	0.0040	0.0065	Main text ref 58
TAHQ/TFM	10	3.30	0.0018	0.0033	Main text ref 59
PI-0.25	10	3.36	0.0017	0.0031	**This work**
PI-0.375	10	3.18	0.0017	0.0030	**This work**
PI-0.5	10	3.29	0.0013	0.0024	**This work**
PI-0.625	10	3.37	0.0015	0.0028	**This work**

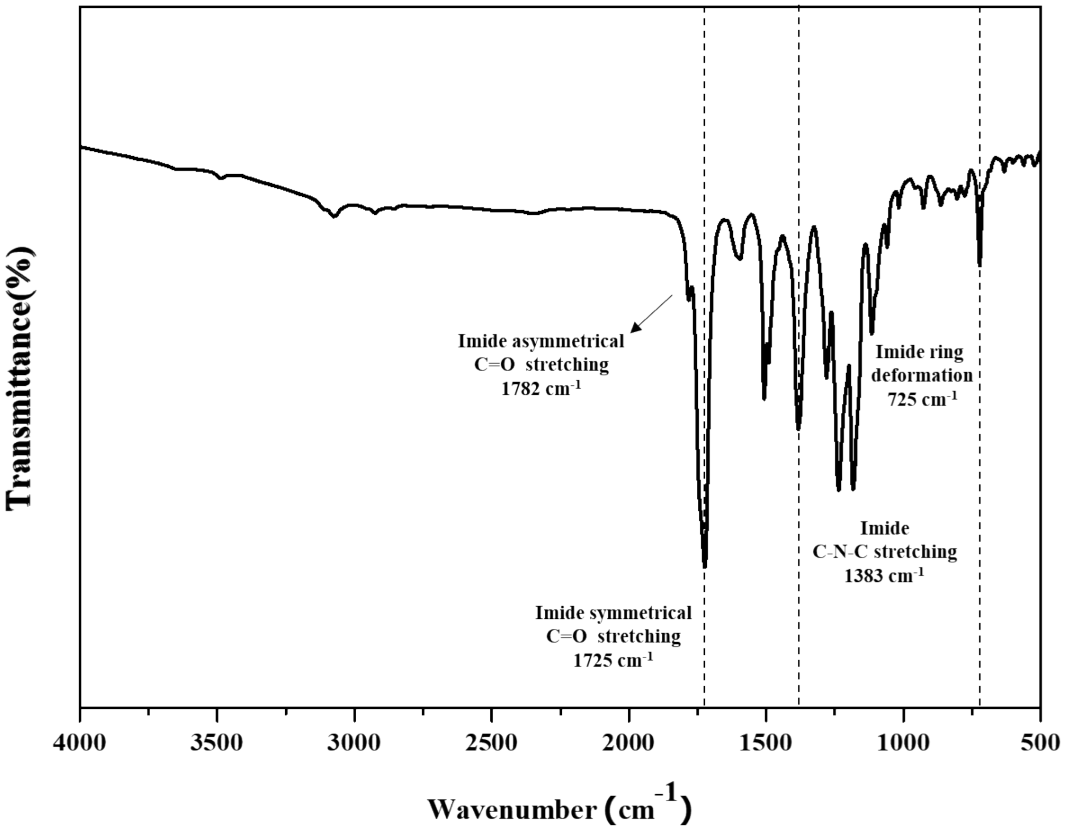

Figure S1. FTIR spectrum of PI-0.5.

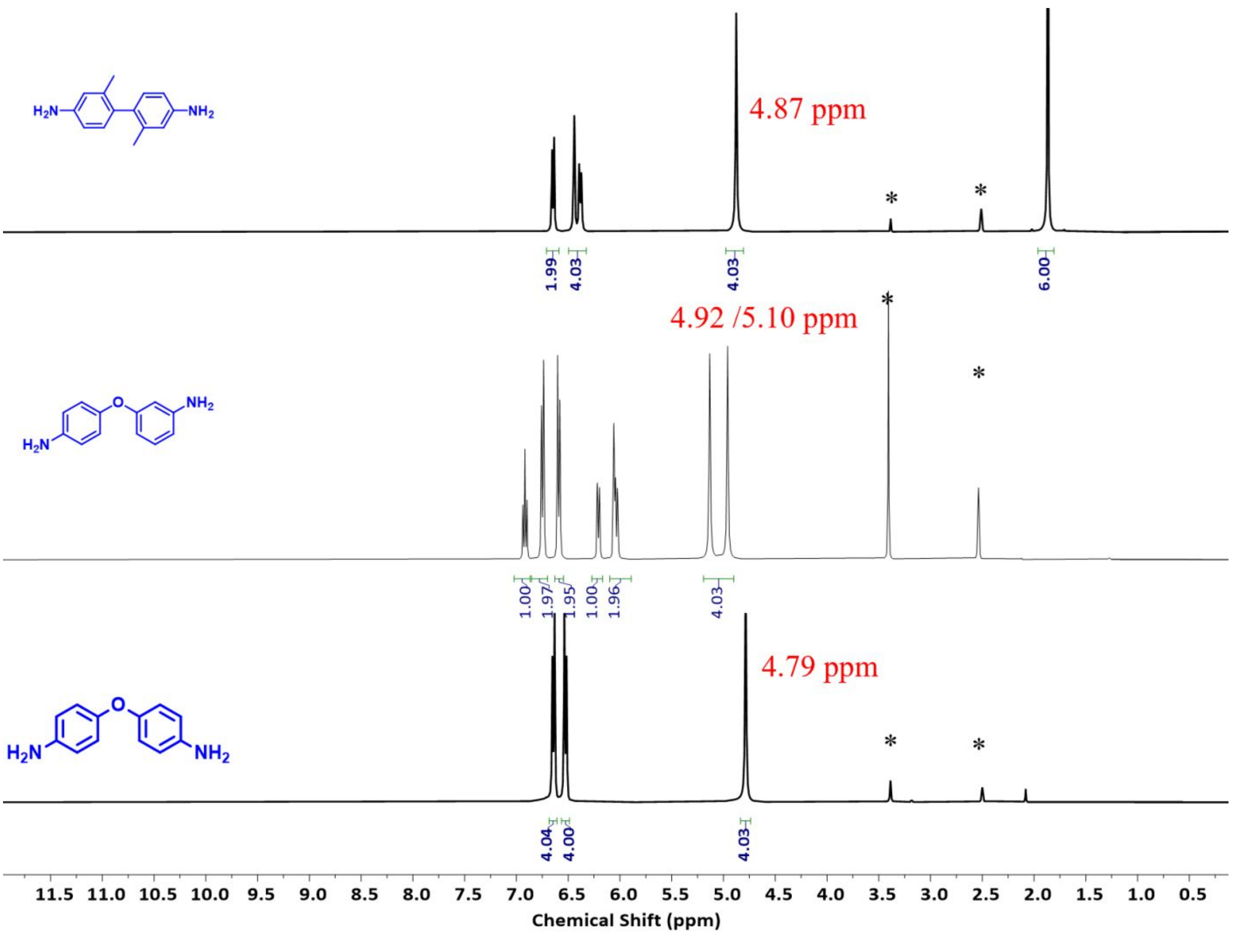

Figure S2. ^{1}H-NMR spectra of m-tolidine, 3,4-ODA, and 4,4-ODA.

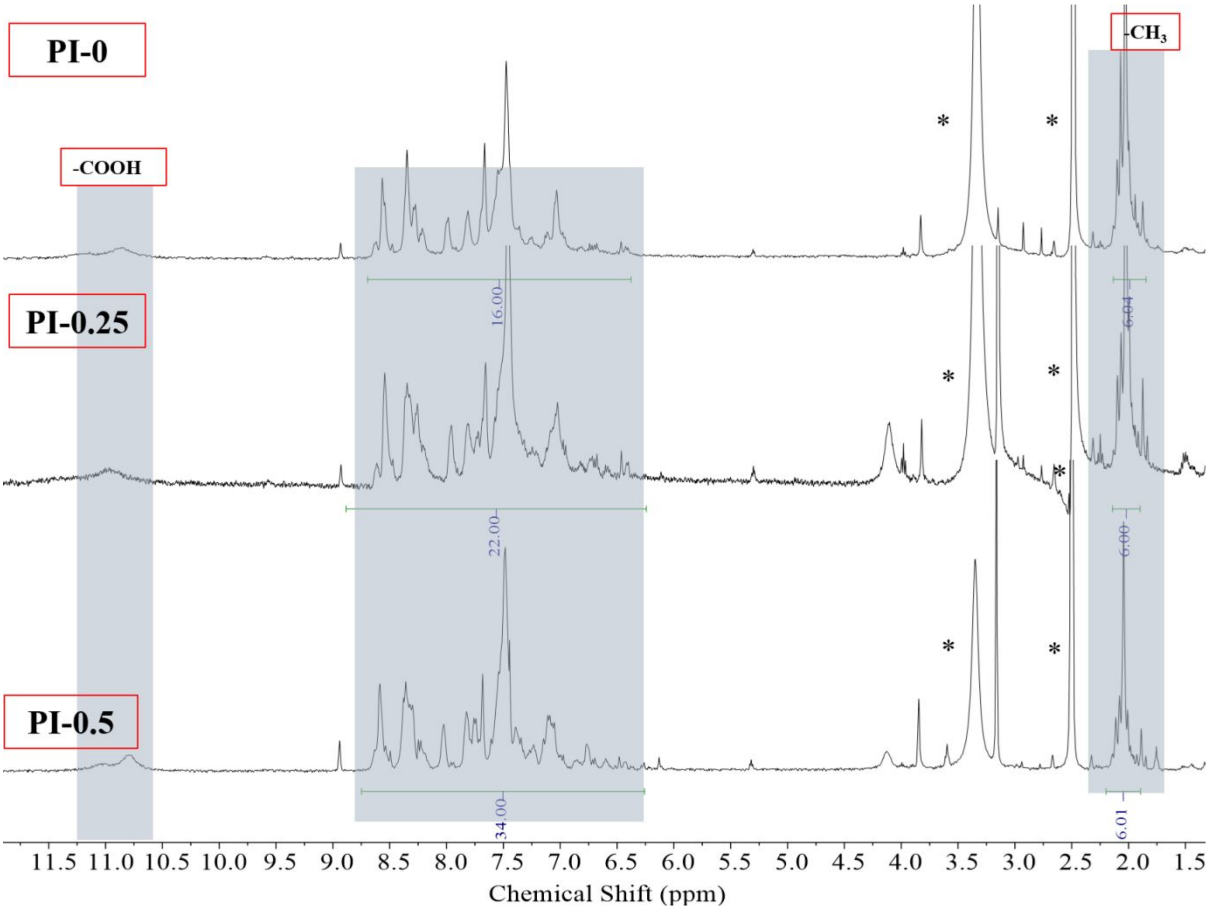

Figure S3. ^{1}H NMR spectra (DMSO-d_6) of PAA precursors for PI-0, PI-0.25, and PI-0.5. Integrals were normalized by setting the m-tolidine methyl peak (two CH_3 groups) to 6.00; the resulting aromatic integrals (16.00, 22.00, 34.00) agree with the theoretical values based on feed composition. Accordingly, the theoretical Ar–H-to-methyl ratios are: 16:6 for PI-0, and (16 x 3/4+18 x 1/4):6 x 3/4=22:6 for PI-0.25, and (16 x 1/2+18 x 1/2):6 x 1/2=34:6 for PI-0.5. These calculated ratios are consistent with the experimental integration results, as shown in Figure S2.

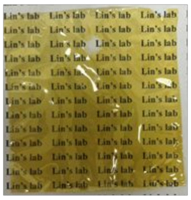	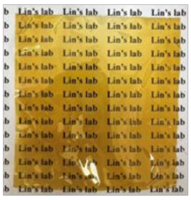	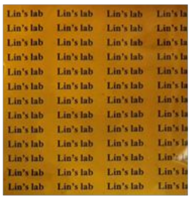	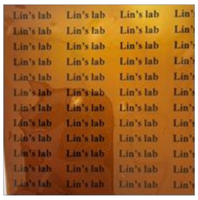
**PI-0**	**PI-0.25**	**PI-0.375**	**PI-0.5**

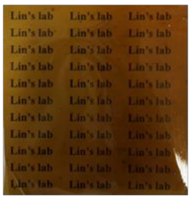	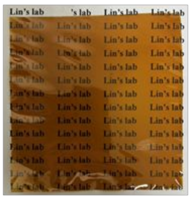	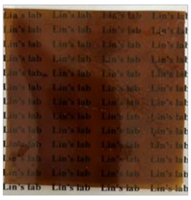
**PI-0.625**	**PI-0.75**	**PI-1**

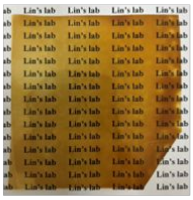	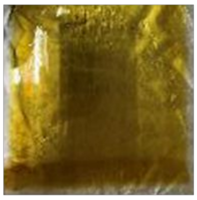
**34-ODA/ODPA**	**44-ODA/TAHQ**

Figure S4. Visual appearance of polyimide films.

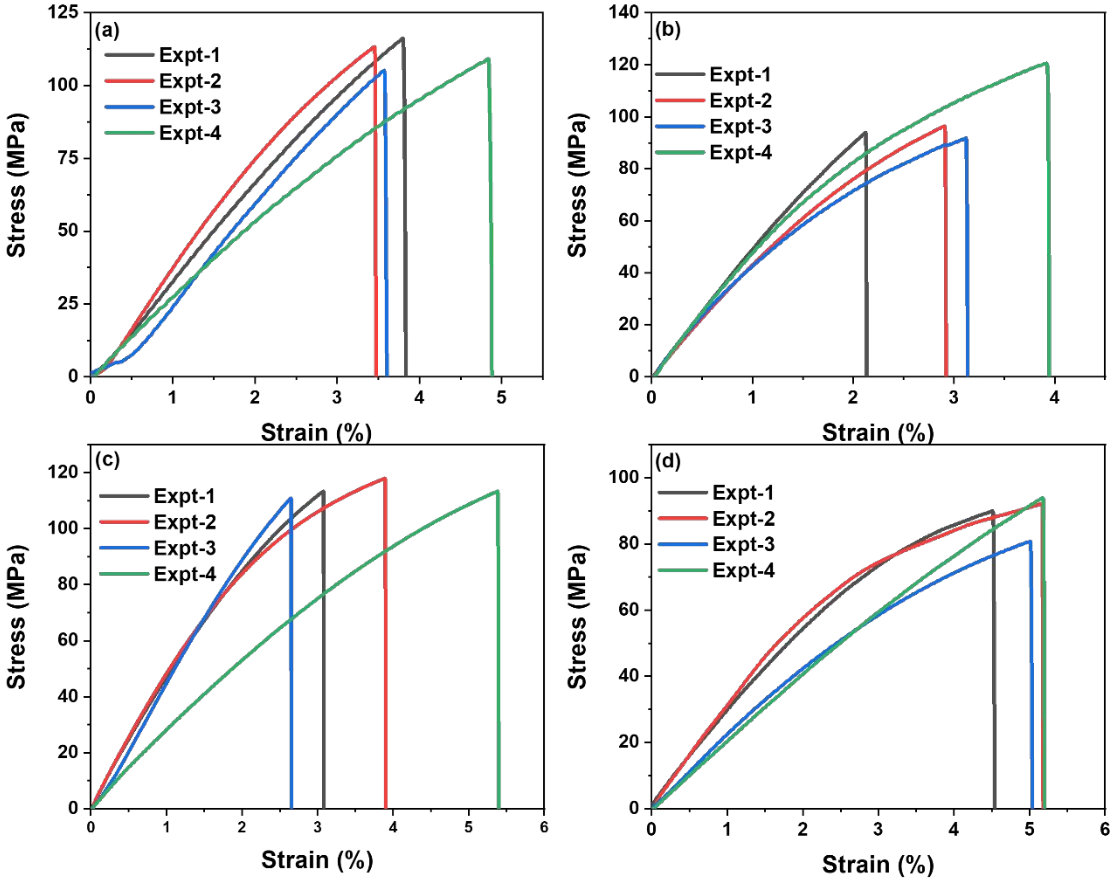

Figure S5. Stress-strain curves of (a) PI-0, (b) PI-0.5, (c) PI-0.625, and (d) PI-0.75 (each sample was measured 4 times).

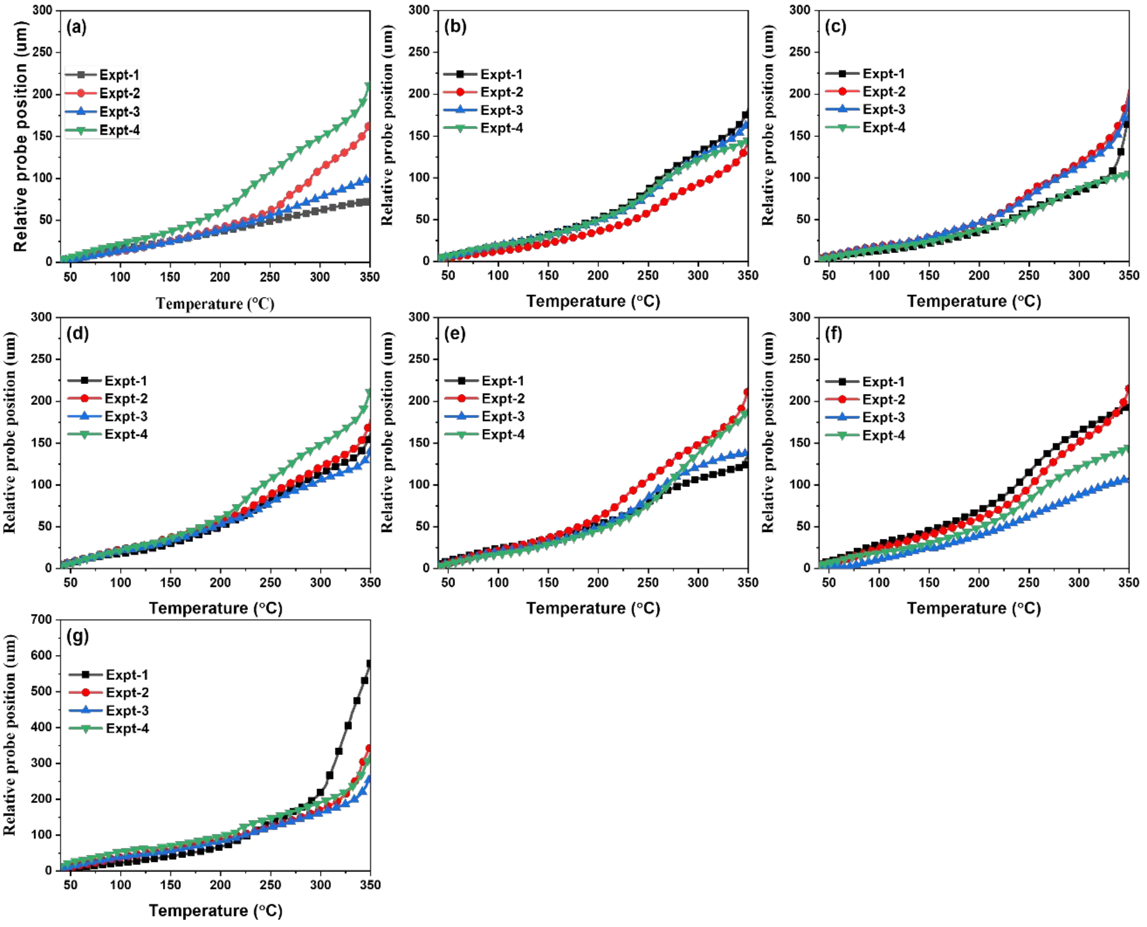

Figure S6. TMA thermograms of (a) PI-0, (b) PI-0.25, (c) PI-0.375, and (d) PI-0.5, (e) PI-0.625, (f) PI-0.75, and (g) PI-1 (each sample measured 4 times).
